# Sensitization of renal carcinoma cells to TRAIL-induced apoptosis by rocaglamide and analogs

**DOI:** 10.1038/s41598-018-35908-0

**Published:** 2018-11-30

**Authors:** Ancy D. Nalli, Lauren E. Brown, Cheryl L. Thomas, Thomas J. Sayers, John A. Porco, Curtis J. Henrich

**Affiliations:** 1 National Cancer Institute, Molecular Targets Program, Frederick, MD 21702 USA; 2 Boston University, Center for Molecular Discovery (BU-CMD), Department of Chemistry, Boston, MA 02215 USA; 3 National Cancer Institute, Cancer Inflammation Program, Frederick, MD 21702 USA; 4 Basic Science Program, Leidos Biomedical Research, Inc., Frederick National Laboratory for Cancer Research, Frederick, MD 21702 USA

## Abstract

Rocaglamide has been reported to sensitize several cell types to TRAIL-induced apoptosis. In recent years, advances in synthetic techniques have led to generation of novel rocaglamide analogs. However, these have not been extensively analyzed as TRAIL sensitizers, particularly in TRAIL-resistant renal cell carcinoma cells. Evaluation of rocaglamide and analogs identified 29 compounds that are able to sensitize TRAIL-resistant ACHN cells to TRAIL-induced, caspase-dependent apoptosis with sub-µM potency which correlated with their potency as protein synthesis inhibitors and with loss of cFLIP protein in the same cells. Rocaglamide alone induced cell cycle arrest, but not apoptosis. Rocaglates averaged 4–5-fold higher potency as TRAIL sensitizers than as protein synthesis inhibitors suggesting a potential window for maximizing TRAIL sensitization while minimizing effects of general protein synthesis inhibition. A wide range of other rocaglate effects (*e*.*g*. on JNK or RAF-MEK-ERK signaling, death receptor levels, ROS, ER stress, eIF4E phosphorylation) were assessed, but did not contribute to TRAIL sensitization. Other than a rapid loss of MCL-1, rocaglates had minimal effects on mitochondrial apoptotic pathway proteins. The identification of structurally diverse/mechanistically similar TRAIL sensitizing rocaglates provides insights into both rocaglate structure and function and potential further development for use in RCC-directed combination therapy.

## Introduction

Induction of cancer cell-specific apoptosis via activation of tumor necrosis factor-related apoptosis-inducing ligand (TRAIL) signaling has been an attractive goal for cancer therapeutics. TRAIL-induced apoptosis is initiated by binding to pro-apoptotic death receptors DR4 and DR5 leading to activation of the extrinsic apoptotic pathway^1–6^ via recruitment and cleavage/activation of pro-caspase 8 in the death-inducing signaling complex (DISC). Activated caspase 8 is released from the DISC and in turn converts pro-caspase 3 to its active form ultimately leading to apoptotic cell death. However, many tumor cells develop resistance to TRAIL-induced apoptosis. Among the most common mechanisms of TRAIL resistance are down-regulation of TRAIL receptors and overexpression of FLICE-inhibitory protein (cFLIP) which competes for binding of procaspase 8 thus blocking its activation^5,6^. Given the relatively common occurrence of TRAIL resistance in many types of cancer as well as non-apoptotic effects of TRAIL^4^, the search for enhancers of TRAIL-induced cell killing has accelerated over the past several years. Death receptor agonists alone or in combination with other agents (including sensitizers) have been used in clinical trials for cancers such as renal cell carcinoma (RCC)^7,8^. Kidney cancer, about 90% of which is RCC, is one of the most common cancer types in the United States and its incidence is increasing (https://www.cancer.org/cancer/kidney-cancer/about.html). However, RCCs display broad resistance against conventional therapies^9^ suggesting a continued need for new therapies. TRAIL initially appeared to be a promising therapeutic approach, but RCCs rapidly develop resistance to TRAIL alone. The proteasome inhibitor bortezomib has been used to sensitize RCC cells to TRAIL, but its use may be limited by toxicity^10^. The therapeutic opportunity for application of TRAIL and TRAIL sensitizers in RCC thus remains attractive. A high-throughput screening (HTS) assay was developed and identified a number of natural product enhancers of TRAIL-induced apoptosis in TRAIL-resistant RCC cells^11–15^. Among the natural products found in the HTS campaign was the protein synthesis inhibitor rocaglamide (ROC) (NSC326408). Protein synthesis inhibitors, including ROC, have previously been identified as able to sensitize various cancer cell lines to TRAIL-induced apoptosis, generally assumed to be due to loss of cFLIP^16–20^.

ROC is a cyclopenta[*b*]benzofuran secondary metabolite isolated from the genus *Aglaia*, with potent antiproliferative, antiviral, antifungal, and anti-inflammatory properties. Structures and biological activities of ROC and analogs (rocaglates) have been the subject of several recent reviews^21–25^. Natural and synthetic rocaglates have been reported to have anticancer activities *in vitro* in various human cancer cell lines and *in vivo* in mouse models. The mechanism of action involved in the anticancer effects of ROC is generally thought to be through inhibition of translation initiation. However, several other cancer-related cellular effects including altered cell cycle progression, RAF-MEK-ERK and p38/JNK signaling, death receptor upregulation, ER stress, generation of reactive oxygen species (ROS), and activation of the intrinsic (mitochondrial) apoptotic pathway have been reported for ROC in various cancer cell types. Many of these cellular effects reported for ROC and analogs have also been demonstrated to sensitize cells to TRAIL-induced apoptosis^1–6^. Due in part to the potential of rocaglates as possible therapeutics for cancer and other diseases, new chemical synthesis methods have been developed and a large number of synthetic rocaglates have been designed for basic studies and pre-clinical development^24–32^. Although advances in synthesis have led to production of both natural rocaglates and novel rocaglamide analogs, few, if any, of these compounds have been investigated for activity as TRAIL sensitizers and neither ROC nor its analogs have been widely assessed in the context of RCC cells.

In order to further investigate the activities and potential for development of rocaglates as TRAIL sensitizers, ROC and 55 natural and synthetic analogs were assessed for their ability to sensitize the well-characterized TRAIL-resistant ACHN RCC cell line to TRAIL-induced apoptosis in parallel with analysis of their protein synthesis inhibitory activity in the same cells under the same conditions. Other previously reported rocaglate effects that are relevant to TRAIL signaling and apoptosis induction were also assessed.

## Results

### Rocaglates sensitize ACHN cells to TRAIL

**Figure 1:**
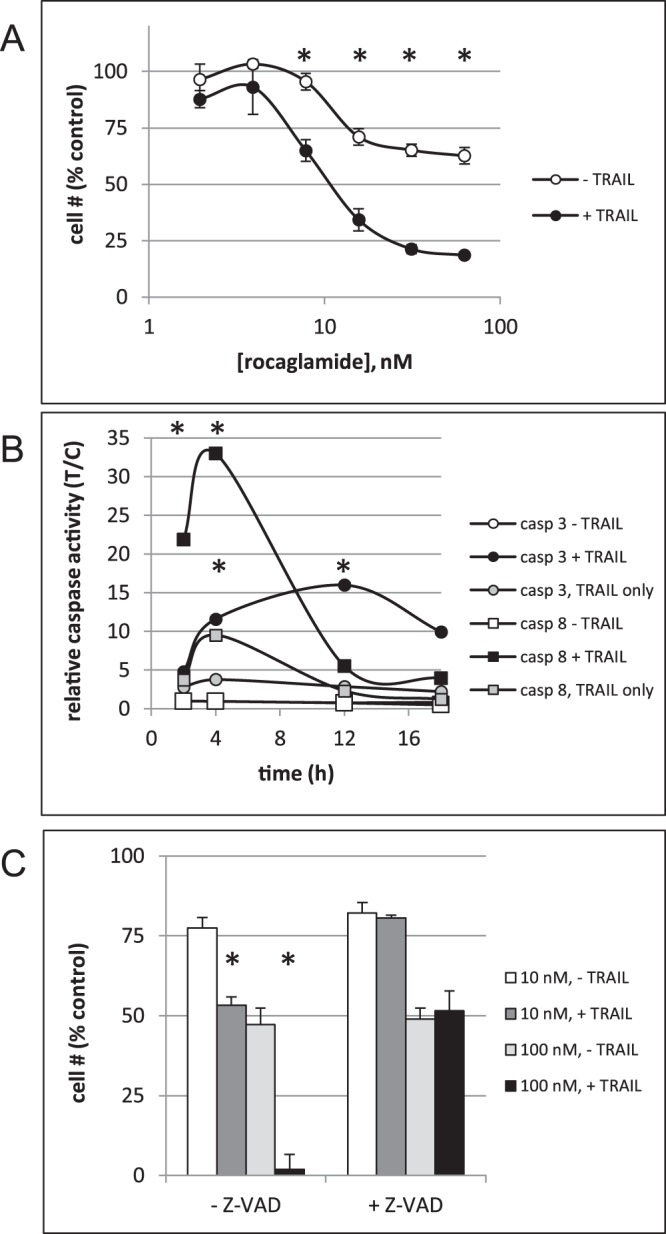
Sensitization of ACHN cells to TRAIL-induced apoptosis by rocaglamide. ACHN renal carcinoma cells (5000/well in 384-well plates) were treated for 4 h with or without various doses of rocaglamide followed by 18 h with or without TRAIL (40 ng/mL). (**A**) Cell survival was estimated by the XTT assay and normalized to untreated control wells. Error bars represent ± sd (n = 3 plates, duplicate wells per plate). *p < 0.001+/− TRAIL. (**B**) Cells were treated for 4 h with 100 nM rocaglamide followed by 2–18 h ± TRAIL and assessed for caspase 3 or caspase 8 activity. Error bars represent ± sd (n = 3) *p < 0.005 compared to control or TRAIL only. (**C**) Cells were pretreated for 2 h with or without 100 µM Z-VADFMK followed by 4 h ± rocaglamide (10 nM or 100 nM final concentration), then ± TRAIL for 18 h and assessment of cell survival by XTT. Error bars represent ± sd (n = 4). *p < 0.005 +/− TRAIL.

**Figure 2:**
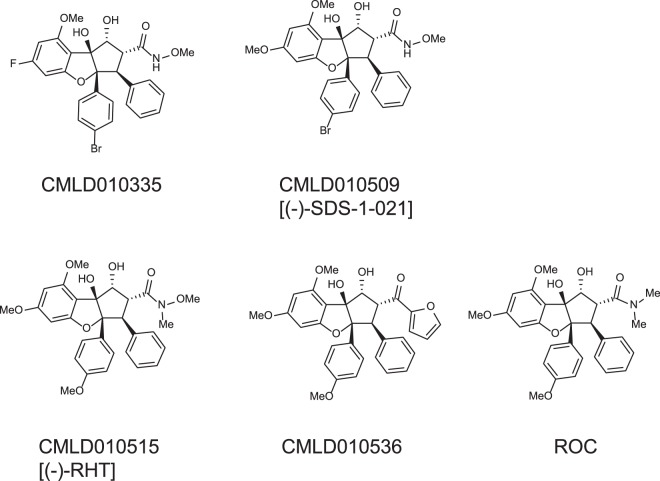
Structures of the highest potency rocaglates (IC_50_ values < 10 nM for TRAIL sensitization) and rocaglamide. Names in brackets correspond to names used elsewhere in the literature (see Discussion).

ROC and analogs (see Supplemental Table S1 for structures) were assessed for their ability to sensitize cells to TRAIL using a previously described assay^11^. The effects of ROC on ACHN cells are shown in Fig. 1. The IC_50_ calculated from repeated dose-response curves for ROC was 28.5 ± 7.5 nM (ave ± sd, n = 15 independent experiments one of which is shown in Fig. 1A). In order to confirm that ROC induced TRAIL-dependent apoptotic signaling, cells were assessed for activation of caspases. Figure 1B demonstrates sequential activation of caspase 8 (death receptor initiator caspase) followed by activation of caspase 3 (effector caspase). Caspase 8 activation in cells pre-treated with ROC was obvious at 2 h after addition of TRAIL and peaked at 4 h whereas caspase 3 activation was maximal ~12 h after addition of TRAIL. The timing of TRAIL-dependent caspase activation was consistent with previous observations with a variety of other TRAIL-sensitizing compounds assessed in ACHN cells^11–13^. Inhibition of caspase activity with ZVAD-FMK eliminated sensitization of the cells to TRAIL-induced apoptosis (Fig. 1C). Taken together, these observations reflect enhanced TRAIL-dependent apoptotic death receptor signaling. In addition to ROC, 28 other rocaglates significantly sensitized these cells to TRAIL – defined as IC_50_ < 1 µM for growth inhibition in the presence of TRAIL (see Supplementary Fig. S1 for dose-response curves for individual rocaglates). The structures of the four most potent TRAIL sensitizers (the only ones with IC_50_ values of <10 nM) along with ROC are shown in Fig. 2. These compounds were also assessed for induction of caspase activity. As with ROC, pre-treatment of cells with these compounds resulted in TRAIL-induced caspase activation and inhibition of sensitization to TRAIL-induced apoptosis by the caspase inhibitor ZVAD-FMK was observed (Supplementary Fig. S2). Although ROC and other rocaglates as single agents resulted in growth inhibition/cytostasis, they did not significantly induce caspase activation (Fig. 1B), even up to 72 h treatment (Supplementary Fig. S2C) nor were their effects as single agents affected by Z-VAD-FMK (Figs 1C and S2B).

### TRAIL sensitization by rocaglates correlates with protein synthesis inhibition and loss of cFLIP protein

**Figure 3:**
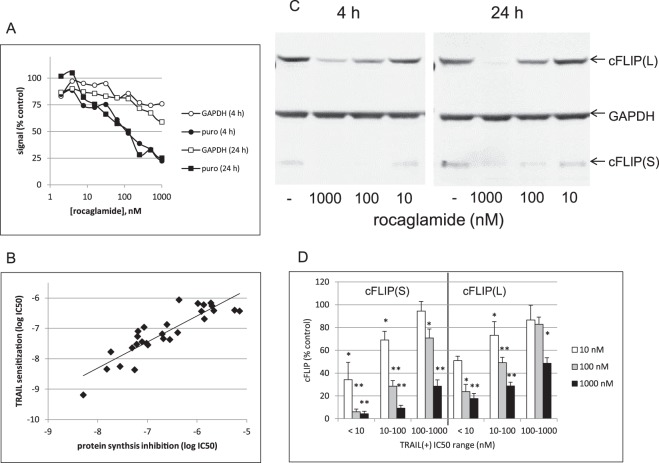
Inhibition of protein synthesis and cFLIP expression by rocaglates. (**A**) ACHN cells in 384-well black wall, clear bottom plates were treated with rocaglamide for 4 h (circles) or 24 h (squares) followed by labeling with puromycin (10 µg/mL final concentration for 30 min), fixation, and processing for ICW analysis of puromycylated protein (see Materials and Methods). (**B**) Correlation of protein synthesis inhibition with TRAIL sensitization for 29 rocaglates active as TRAIL sensitizers (*i*.*e*., <1000 nM IC_50_). Best fit line shown; slope: 0.86, correlation coefficient: 0.88. (**C**) ACHN cells were treated for 4 h or 24 h with 10, 100, or 1000 nM rocaglamide followed by analysis of cFLIP protein expression. (**D**) Effects of the 29 active rocaglates on cFLIP protein expression by ACHN cells were individually assessed by quantitative western blot analysis. Results were binned according to IC_50_ values for sensitization to TRAIL. cFLIP values (normalized to GAPDH and presented as % of untreated control values) were averaged for all compounds in each bin. Cells were treated 4 h with rocaglates alone at 10 nM, 100 nM, or 1000 nM. Error bars represent sem, n = 4–13. See Supplementary Table S1 for protein synthesis inhibition, Supplementary Figure S3 for cFLIP expression data for individual rocaglates and Supplementary Fig. S10 for full-length cFLIP/rocaglamide blot. *p < 0.05, **p < 0.001 compared to untreated control.

The rocaglates were assessed for their ability to inhibit protein synthesis in ACHN cells by quantitation of incorporation of puromycin into newly synthesized protein after treatment with compounds^20^. Figure 3A shows the dose-dependent inhibition of protein synthesis by ROC. There was no significant difference between effects after 4 h vs. 24 h treatment. Protein synthesis inhibition by the remaining rocaglates was measured after 4 h incubation. IC_50_ values for protein synthesis inhibition and for TRAIL sensitization for the all of the rocaglates, sorted by structural class, are shown in Supplementary Table S1. These data were used to compare potencies of rocaglates as TRAIL sensitizers and as protein synthesis inhibitors and revealed a clear correlation (Fig. 3B). In general, the rocaglates were more potent as TRAIL sensitizers than as protein synthesis inhibitors under these experimental conditions (IC_50_s for protein synthesis inhibition averaged 4–5 x IC_50_s for TRAIL sensitization). Since other protein synthesis inhibitors have been shown to sensitize various cells (including ACHNs) to TRAIL-induced apoptosis *via* down-regulation of cFLIP, a quantitative western blot assay was used to determine the effects of the rocaglates on protein expression of cFLIP. Figure 3C shows representative western blot analysis of cFLIP after ROC treatment. Quantitation of the long and short isoforms of cFLIP after treatment by ROC and the other TRAIL-sensitizing rocaglates showed general correlation between cFLIP protein levels and TRAIL sensitization activity (Fig. 3D). The entire quantitative cFLIP expression data set for the active rocaglates is shown in Supplementary Figure S3. In general, the cFLIP_S_ isoform appears to be more sensitive to rocaglates than the long form. ROC did not affect cFLIP mRNA levels (Supplementary Fig. S9).

### Effects of rocaglates on other apoptotic targets

**Figure 4:**
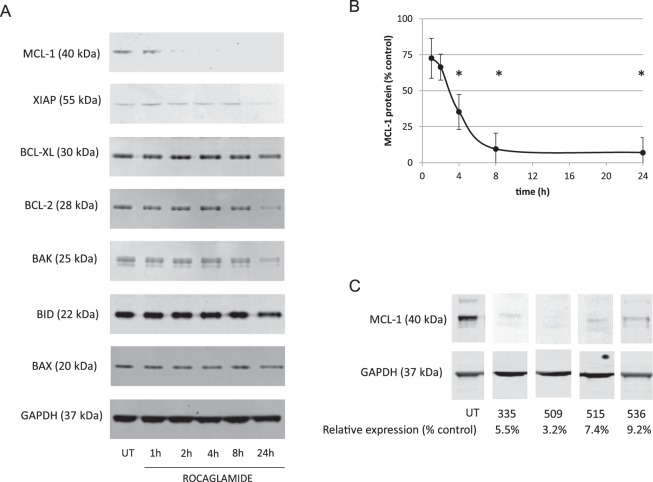
Rocaglates affect expression of MCL-1. (**A**) ACHN cells were treated with rocaglamide for 1–24 h followed by western blot analysis of indicated pro- and anti-apoptotic proteins. (**B**) Quantitative western blot analysis of ROC-treated cells. *p < 0.05 compared to untreated control. (**C**) Quantitative western blot analysis of MCL-1 protein assessed after 4 h treatment with the indicated rocaglate. Quantitative values were normalized to loading control (GAPDH) and expressed as a % of signal for untreated cells run on the same gel. MCL-1/GAPDH ratios shown in Supplementary Fig. S10. Cropped blot images are shown – see Supplementary Fig. S10 for full-length blots.

Changes in expression of some BCL-2 family mitochondrial proteins implicated in apoptosis have also been reported after ROC treatment. Other than a slight reduction in some cases after 24 h, BAK, BAX, BCL-2, BCL-XL, and BID levels were unchanged. Similarly, XIAP levels were not affected except for a small decrease after 24 h. However, MCL-1 was diminished after treatment with ROC (Fig. 4A,B) or the four most active rocaglates (Fig. 4C).

### ROC treatment leads to cell cycle arrest

**Figure 5:**
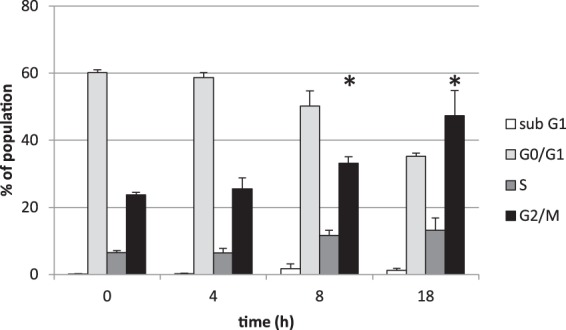
Rocaglamide treatment leads to cell cycle arrest. ACHN cells were treated with 1000 nM rocaglamide for the indicated times followed by flow cytometry analysis of cell cycle. Error bars represent sd. *p < 0.05 for accumulation compared to control.

All of the active rocaglates also inhibited cell growth by approximately 50% as single agents at up to 10 µM (Supplemental Fig. S1). Given that the doubling time for ACHN cells under these experimental conditions is ~24 h, the rocaglate-induced growth inhibition was probably a cytostatic effect rather than cell death. ROC treatment resulted in cell cycle arrest at G2/M (Fig. 5). However, neither ROC nor 9 other rocaglates representing different structural classes (all < 50 nM IC_50_ for TRAIL sensitization) induced apoptosis (<2-fold increase in caspase 3 activity after up to 72 h treatment with 1 µM – Supplementary Fig. S2C).

### Effects of rocaglates on other targets

**Figure 6:**
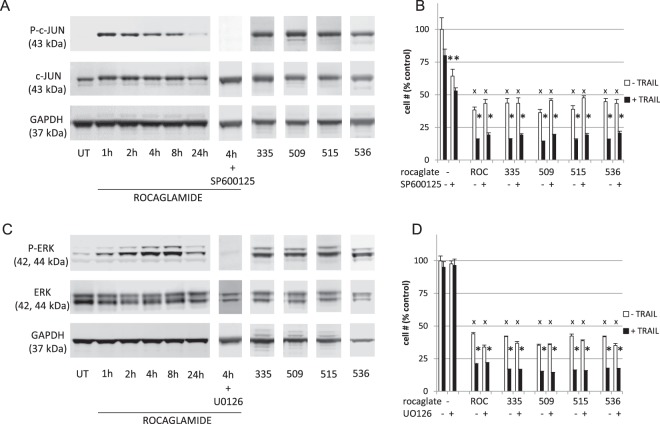
Effects of rocaglates on JNK and MEK/ERK signaling. ACHN cells were treated with rocaglamide for 1–24 h, or the indicated rocaglate for 4 h followed by western blot analysis. In parallel, prior to rocaglamide addition, cells were pre-incubated for 1 h with the indicated kinase inhibitor. (**A**) phosphoJUN, (**C**) phosphoERK. For panels B (JNK inhibitor) and D (MEK inhibitor), cells were pretreated for 1 h with or without inhibitor as indicated followed by rocaglate (100 nM) for 4 h, then with (black bars) or without (open bars) TRAIL overnight followed by XTT assay (cell numbers were normalized to untreated wells and shown as % of untreated control ± sd). Statistical significance: *p < 0.01 (TRAIL+ vs TRAIL− pairs), **p < 0.01 (SP600125− vs. SP600125+), ^x^p < 0.01 (rocaglate− vs. rocaglate+). Cropped blot images are shown – see Supplementary Fig. S10 for full-length blots.

ROC and other rocaglates have been reported to affect a number of cellular phenomena that have also been reported to affect sensitivity of cells to TRAIL. Among these are effects on JNK and RAF-MEK-ERK signaling, ROS generation, and ER stress. Rapid activation by ROC of JNK (as measured by SP600125-sensitive JUN phosphorylation) and MEK/ERK signaling (as measured by UO126-sensitive increase in phosphoERK) was observed and the 4 most potent rocaglates had similar effects (Fig. 6). Neither total JUN nor total ERK levels were affected by any of the rocaglate treatments. Pretreatment with UO126 or with SP600125 did not alter either TRAIL-dependent or TRAIL-independent effects on cell numbers (Fig. 6 panels B and D). ROS generation was not detected (data not shown) and N-acetyl cysteine (NAC) pretreatment had no effect on cell numbers ± TRAIL. Supplementary Fig. S4 demonstrates that inhibition of MEK/ERK signaling, JNK signaling, or ROS generation by UO126, SP600125, and NAC respectively did not eliminate the TRAIL sensitizing effects of these or a selection of other rocaglates representing a range of structural classes. CHOP, an indicator of ER stress did not increase upon ROC treatment (data not shown). Levels of TRAIL receptor DR4, prohibitins, phosphoITCH, and eIF4E protein levels were also unaffected by ROC treatment and DR5 was slightly reduced. eIF4E phosphorylation was transiently increased, but substantially reduced by 24 h (Supplementary Fig. S5). Surface levels of TRAIL receptors were also analyzed by flow cytometry (Supplementary Fig. S6). DR4 levels did not change, nor did levels of cell surface “decoy” receptors (TR3 and TR4). Interestingly, however, unlike total DR5, surface DR5 expression appeared to increase with ROC treatment.

### Effects of rocaglates on other renal cells

**Figure 7:**
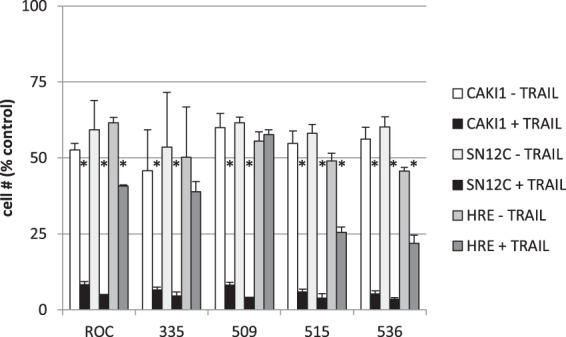
Effects of rocaglates on CAKI-1, SN12C, and HRE. Renal carcinoma or HRE cells (5000/well in 384-well plates) were treated for 4 h with or without the indicated rocaglate followed by 18 h with or without TRAIL (40 ng/mL). Cell survival was estimated by the XTT assay and normalized to untreated control wells. Error bars represent ± sd (n = 4). *p < 0.01 (TRAIL+ vs. TRAIL− pairs). Complete dose-response curves are shown in Supplementary Figs S7 and S8.

ROC and the four most potent analogs all sensitized CAKI-1 and SN12C RCC cell lines to TRAIL-induced apoptosis in parallel with inhibition of protein synthesis in these cells (Fig. 7 and Supplementary Fig. S7). Relative potency of the 5 compounds followed that observed with ACHN cells for both measurements (Supplementary Fig. S7). Protein synthesis in human renal epithelial (HRE) cells was also affected at similar potencies, but HRE cells were considerably less susceptible to TRAIL sensitization (Figs 7 and S8).

## Discussion

A series of 29 structurally diverse rocaglates, including ROC, were identified as potent sensitizers of TRAIL-induced apoptosis in ACHN renal carcinoma cells. Pretreatment with these rocaglates primed the cells to respond to TRAIL. Protein synthesis inhibition by rocaglates resulted in rapid loss of cFLIP protein leading to enhanced death receptor signaling and subsequent caspase-dependent apoptotic cell death upon addition of TRAIL. Upregulation of TRAIL receptors is a common contributor to sensitization of cells to TRAIL-induced apoptosis and has been reported in response to ROC in other cell systems, but neither total DR4 nor DR5 were upregulated by ROC. Similarly, surface expression of “decoy” TRAIL receptors could contribute to TRAIL resistance, but ROC did not affect these levels. However, cell surface DR5 did increase. Loss of cFLIP alone has been shown to sensitize ACHN cells to TRAIL^16^. Interestingly, the rocaglates were several fold more potent as TRAIL sensitizers as compared to protein synthesis inhibition (in parallel experiments under the same conditions) suggesting that complete inhibition of cFLIP synthesis may not be necessary for TRAIL sensitization. cFLIP degradation is regulated by the JNK/ITCH pathway^6^ and JNK was activated by rocaglates, as has been previously reported in other cell systems^21^. JNK phosphorylates and activates ITCH, the ubiquitin ligase that controls cFLIP degradation^6,33,34^. However, ROC treatment did not affect levels of total or phospho-ITCH nor did inhibition of JNK affect TRAIL sensitization. cFLIP levels are regulated by transcription, translation, and degradation. Given that ROC did not affect cFLIP mRNA levels or ITCH activation, it is likely that the effects of the rocaglates on cFLIP are due to translation inhibition although other mechanisms cannot be entirely ruled out. These observations are consistent with the conclusion that inhibition of protein synthesis rather than increased proteasomal degradation probably results in rocaglate-induced loss of cFLIP proteins. Targets of translational inhibition by rocaglates are, in part, defined by their 5′ UTRs and this may lead to specific effects on cFLIP and potential cFLIP regulatory proteins^22,24^. Cross-talk between the intrinsic (mitochondrial) and extrinsic (death receptor) apoptotic pathways can result in diminished or amplified apoptotic signaling and the balance of pro- and anti-apoptotic mitochondrial proteins can significantly affect TRAIL-induced apoptosis^35^. In particular, XIAP overexpression can contribute to TRAIL resistance and increased expression of BAX and BAK can increase sensitivity to TRAIL. Other than MCL-1, however, the other mitochondrial proteins assessed did not change significantly by up to 8 h treatment with ROC and are thus probably not major contributors to predisposing ACHN cells to respond to subsequent treatment with TRAIL. After 24 h treatment with ROC, the result is more complicated in that there are both pro-apoptotic (BAK) and anti-apoptotic (BCL-2 and XIAP) proteins that were diminished whereas BID and BAX (pro-apoptotic) and BCL-XL (anti-apoptotic) were essentially unchanged. Investigation of rocaglate-induced interactions between the intrinsic and extrinsic pathways would be of interest in future studies as would possible cell type-specific effects (*e*.*g*. rocaglates as single agents did not induce apoptosis in ACHN cells). The rapid loss of MCL-1 protein upon rocaglate treatment may enhance the TRAIL-sensitization effect of the parallel loss of cFLIP protein. MCL-1 can cooperate in sensitization of cells to TRAIL^35^. However, as seen with other RCC cell lines^36^, knockdown of MCL-1 with siRNA did not sensitize ACHN cells to TRAIL (data not shown) suggesting that loss of MCL-1 may play only a minor role, if any, in sensitization of ACHN cells to TRAIL by rocaglates. Increased cell surface expression of DR5, on the other hand, may contribute to increased TRAIL signaling.

Induction of cell cycle arrest by ROC and analogs has been reported to occur at G1/G0 or at G2/M (with insignificant cell death) depending on the cells investigated^21,24,37^. In this study with ACHN cells, treatment with ROC alone led to G2/M cell cycle arrest without subsequent apoptotic cell death. Reported effects of rocaglates on RAF-MEK-ERK signaling have also been cell-type-specific. RAF-MEK-ERK signaling has often been reported to be inhibited (often involving prohibitin) leading to CDC25A degradation and G1 cell cycle arrest and loss of eIF4E phosphorylation^21^, but sometimes reported to be increased. For example, in Jurkat cells ERK phosphorylation was inhibited by ROC and analogs while ERK phosphorylation was significantly increased in NIH/3T3 cells under the same conditions^38^. Although ERK activation typically drives cell cycle progression, it can result in G2/M arrest^39,40^. However, given that ERK activation was transient and inhibition affected neither the cytostatic effects of rocaglates nor their TRAIL sensitization effects, the functional significance of enhanced RAF-MEK-ERK signaling in ROC-treated ACHN cells is uncertain. Similarly, eIF4E phosphorylation, which results from RAF-MEK-ERK pathway activation^21,22,38^, was transiently increased by rocaglamide treatment (peaking at 4–8 h) followed by reduction to less than control levels by 24 h. However, translation inhibition was persistent from 4–24 h as were reductions in the levels of cFLIP and MCL-1. These results are consistent with the previous conclusion that inhibition of translation by rocaglates is independent of eIF4E phosphorylation^38^.

Four of the rocaglates, three of which were hydroxamates, were considerably more potent than ROC itself (<10 nM) and one compound (SDS-1–021) had sub-nM potency as a TRAIL sensitizer (see Supplementary Table S1). A number of the compounds assessed in this work have previously been investigated in other cell systems. In particular, hydroxamates^27^ such as SDS-1-021 (CMLD010509), (−)-RHT (CMLD010515), and CR-1–31B (CMLD005522) have been used in a variety of applications^38,41–44^. In previous studies, SDS-1-021 was typically the most potent of the rocaglates investigated as also found here. For example, Chu, and coworkers^38^ found it to be more potent than ROC which was in turn more potent than CR-1-31B in Jurkat cells (CR-1-31B and SDS1-021 were equipotent in NIH-3T3 cells) with low nM IC_50_s. In the present study, in general, substituting hydroxamates for the amide of ROC maintains or enhances potency while conversion to ester decreases potency (*e*.*g*., compare ROC to CMLD010852). Other isosteric substitutions for the amide or its elimination were generally well-tolerated (*e*.*g*., compare ROC to CMLD010536 or to (±)-rocaglaol). Substitution of the methoxy group on ring B of the molecule (nomenclature per Pan, and coworkers^22^) significantly increases potency (*e*.*g*., compare CR-1-31B with SDS-1-021). As seen with ACHN cells in the present report, *exo-*RHT (CMLD010441), unlike the bioactive *endo* stereoisomer (−)-RHT, is a poor protein synthesis inhibitor^28^. In fact, all *exo* diasteroisomers of active compounds that were tested were either inactive or had very low (>0.5 µM) activity with TRAIL sensitization and >1 µM for protein synthesis inhibition (see Supplementary Table S1). The tested aglains and rocaglate β-lactones were inactive.

Although the rocaglates were generally more potent as TRAIL sensitizers than as protein synthesis inhibitors, there were a few exceptions (Supplementary Table S1). In particular, three compounds in the oxazinone/pyridazone ring system class (CMLD010535, 506, and 483) and one with a monofluorinated phloroglucionol ring (CMLD010337) were roughly equipotent. The one thiazole ketone tested (CMLD005551) was slightly more potent as a protein synthesis inhibitor, although it was one of the least potent of the active compounds. These outliers may suggest possible alternative targets in addition to protein synthesis inhibition that may merit further investigation.

Rocaglamide and its analogs have been extensively investigated. As a result, a significant amount is known about their general cell biological and biochemical effects. In particular with regard to TRAIL, there are a number of reported effects of rocaglates that have also (in other contexts) been demonstrated to be able to sensitize cells to TRAIL. Among these are ROS generation, MEK/ERK signaling, JNK signaling (including ITCH effects), death receptor expression levels, activation of the intrinsic mitochondrial pathway, ER stress, cell cycle arrest. All of these effects have been addressed in the present work conclusively ruling out a large number of otherwise plausible mechanisms. This leads inevitably to the conclusion that protein synthesis inhibition leading to loss of cFLIP, potentially augmented by loss of MCL-1 and increased cell surface DR5, result in sensitization of TRAIL-resistant RCC cells to TRAIL-induced apoptosis.

ROC and analogs continue to be pursued as stand-alone anti-cancer agents. In particular, rocaglates are being investigated as potential therapeutics for a variety of cancer types including leukemias, melanomas, myelomas, lung cancer, breast cancer, colon cancer, prostate cancer and glioblastoma due to their multiple mechanisms of action; induction of apoptosis via protein synthesis inhibition, modulation of signaling pathways, cell cycle regulation, etc.^21,23^. The results reported here suggest expansion of these potential applications to include combination therapies (with TRAIL) for RCC as well as providing additional insights into their actions and structure-activity relationships. Although beyond the scope of this investigation, these results also illustrate a clear need to develop contextual understanding of the effects of rocaglates on various signaling pathways and the consequences of those effects in cellular context. As has been seen in the past, there are important differences and similarities both in regard to TRAIL sensitization mechanisms and effects as stand-alone reagents depending on the cellular environment of the study which will be important future investigative pathways.

## Materials and Methods

### Chemicals and reagents

2,3-*Bis*(2-methoxy-4-nitro-5-sulfophenyl)-5-[(phenylamino)carbonyl]-2H-tetrazolium hydroxide (XTT; NSC 601519) was obtained from the NCI Drug Synthesis and Chemistry Branch. Sources of other reagents included: recombinant TRAIL ligand (168 amino acid TNF homologous extracellular domain – Peprotech, Rocky Hill, NJ); Z-VAD-FMK (Promega, Madison, WI); cell culture media and additives (Cellgro, Hyclone, Sigma, or Invitrogen); BCA protein assay kits (Pierce/Thermo); puromycin (Invitrogen, Carlsbad, CA). Monoclonal mouse anti-puromycin clone 12D10 and rabbit anti-GAPDH (ABS16) were obtained from EMD-Millipore (Billerica, MA). Secondary antibodies: Goat anti-rabbit (800) and Goat anti-mouse (680) and blocking buffer were from LiCor (Lincoln, NE). Other reagents from Sigma (St. Louis, MO).

### Sources of rocaglates

ROC (NSC326408) and didesmethyl rocaglamide (NSC705956) were provided by the National Cancer Institute (Frederick, MD). The remaining rocaglates were from the Boston University Center for Molecular Discovery (BU-CMD) compound collection.

### Cell growth and apoptotic signaling assays

ACHN, CAKI-1, and SN12C cells (NCI) were maintained and growth was assayed as described^11,13^. Human renal epithelial (HRE) cells were purchased from Lonza (Walkersville, MD) and maintained according to the company’s recommendations. For TRAIL sensitization assays, cells were allowed to attach overnight (3500–5000 cells/well, 384-well plates) followed by 4 h with compounds or DMSO. TRAIL (40 ng/ml final) was added and cell numbers were estimated (18–24 h) using XTT. For analysis of reactive oxidative species (ROS) involvement, N-acetyl cysteine (NAC, 10 mM) was added just before compounds and remained in the plates throughout. NAC interference with the XTT assay was previously observed^12^, so cell numbers were estimated by measuring total cell protein using Sulforhodamine B^11^. For caspase inhibition, cells were pretreated with 100 µM ZVAD-FMK. For assessment of caspase activation, cells were treated for 4 h followed by TRAIL (1–24 h). For assessment of caspase 3 activation in the absence of TRAIL, cells were treated with rocaglate for up to 72 h. Assessment of caspase 8 or 3 utilized Caspase-Glo^TM^ 8 or 3/7 assay kits (Promega) according to manufacturer’s instructions. The use of these kits for evaluation of caspase activation has been previously validated for ACHN cells^11–13^.

### Quantitation of protein synthesis

Protein synthesis was assessed by measuring incorporation of puromycin to quantitate nascent polypeptides in an InCell western (ICW) format as described^20^. Briefly, cells plated in black-wall, clear-bottom, polylysine-coated plates were treated with indicated compounds followed by pulse-labeling with puromycin (10 µg/mL for the final 30 min of the treatment). After formaldehyde fixation, permeabilization (0.1% Triton X-100 (v:v) in PBS), extensive washing, and blocking (LiCor blocking buffer), mouse anti-puromycin and rabbit anti-GAPDH were mixed and added. Secondary antibodies were goat anti-mouse (680) and goat anti-rabbit (800) (from LiCor) to detect puromycin and GAPDH respectively. For quantitation, plates were scanned on the LiCor Odyssey instrument in “InCellWestern” mode capturing relative fluorescence in each channel.

### Immunoblot

Cells (2 × 10^6^ cells/well, 6-well plates) were treated and extracted for SDS-PAGE and immunoblot analysis. The following primary antibodies were used: Monoclonal mouse anti-cFLIP clone 7F10 was obtained from Enzo (Farmingdale, NY). Polyclonal rabbit anti-MCL-1, anti-BCL- xL, anti-BCL-2, anti-BID, anti-Bak, anti-Bax, polyclonal rabbit anti-c-JUN, anti-phospho-c-JUN (Ser 63) II, anti-p44/42 MAPK (Erk1/2), monoclonal rabbit anti-phospho-p44/42 MAPK (ERK1/2) (Thr202/Tyr204) clone 197G2, anti-DR4 clone D9S1R, anti-DR5 clone D4E9, polyclonal rabbit anti-eIF4E and anti-phospho eIF4E (Ser209) were obtained from Cell Signaling Technologies (Beverly, MA). Polyclonal rabbit anti-prohibitin was obtained from Abcam (Cambridge, MA). Primary antibodies for target proteins and loading control (GAPDH) were paired such that one was murine and the other rabbit. Detection employed secondary antibodies labeled with near IR fluorophores (same as in ICW). Blots were scanned using the LiCor Odyssey scanner and each channel simultaneously visualized and quantitated using the instrument software^20^. Cropped gray-scale images of blots are shown in the main text figures while full-length color images are shown in Supplementary Information for all of the blots from which the figures were derived.

### Cell cycle analysis

ACHN renal carcinoma cells (1.5 × 10^6^/well in 6-well plates) were treated with DMSO (control) or ROC (1000 nM) and incubated for 4, 8 or 18 h. After incubation, the cells were collected by trypsinization and fixed in 100% ice cold methanol for 15 minutes. Fixed cells were decanted and washed with PBS, resuspended in 150 μL of Propidium Iodide staining solution (containing propidium iodide, RNase and PBS) and then incubated at 37 °C for 40 minutes. Stained cells were collected by centrifugation, washed and resuspended in 200 μL PBS, and visualized using Cellometer Vision CBA image cytometer. Stained cells were analyzed for cell cycle distribution using Vision CBA and FCS Express Software. Results were binned per cell population in different phases of cell cycle (represented as % of total cell population).

### Data analysis and presentation

Unless otherwise noted, all results were normalized to control (DMSO)-treated cells and quantitation reported as average ± standard deviation. Statistical significance of cell survival in the presence vs. absence of TRAIL was assessed by t-test (p < 0.05 considered significant). IC_50_ values were estimated from dose-response curves using SigmaPlot 4-parameter logistic nonlinear regression analysis (SPSS, Inc.). Quantitation of western blot data and ICW (*i*.*e*., puromycylation) data included normalization of signals to GAPDH.

## Electronic supplementary material

Supplementary Information

## Data Availability

The data contributing to this study are included in the manuscript and/or Supplementary Information.
