# Supplementary material for: Sensitization of renal carcinoma cells to TRAIL-induced apoptosis by rocaglamide and analogs
Source: Sci Rep. 2018 Nov 30;8:17519. doi: 10.1038/s41598-018-35908-0 (PMC6269514; doi:10.1038/s41598-018-35908-0)

SUPPLEMENTARY INFORMATION

Sensitization of renal carcinoma cells to TRAIL-induced apoptosis by rocaglamide and analogs

Nalli, AD, Brown, LE, Thomas, CL, Sayers, TJ, Porco, JA Jr., Henrich, CJ

Contents:

page 2: Supplementary Table S1: Rocaglate structures, TRAIL sensitization, and protein synthesis inhibition

page 12: Supplementary Figure S1: Effects of rocaglates on ACHN growth/survival

page 16: Supplementary Figure S2: Effects of the 4 most potent rocaglates on death receptor signaling

page 18: Supplementary Figure S3: Effects of the rocaglates on cFLIP protein expression

page 19: Supplementary Figure S4: Rocaglate effects are unaffected by inhibition of MEK, JNK, or ROS

page 20: Supplementary Figure S5: Rocaglamide effects on selected other proteins

page 21: Uncropped blots for Figure S5

page 23: Supplementary Figure S6: Surface expression of TRAIL receptors

page 24: Supplementary Figure S7: Effect of rocaglates on CAKI-1 and SN12C cells

page 26: Supplementary Figure S8: Effect of rocaglates on HRE cells

page 27: Supplementary Figure S9: Rocaglamide does not affect expression of cFLIP mRNA

page 28: Supplementary Figure S10: Original uncropped blots – data for Figures 3, 4, and 6 in main text

SUPPLEMENTARY TABLE S1

TRAIL sensitization and protein synthesis inhibition for all tested rocaglates (sorted by TRAIL sensitization potency within each structural class)

**S1.1 Amides and Hydroxamates**			*IC_{50} (nM)*	
Designation (used in pub)	*Other Name(s)*	*Structure*	*TRAIL*	*protein synthesis*
CMLD010509 (racemic)	SDS-1-021	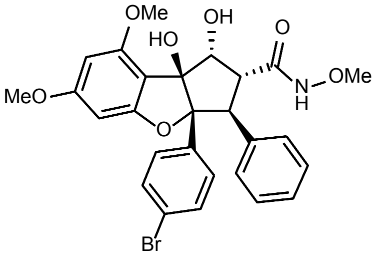	0.6	5.1
CMLD010335 (racemic)		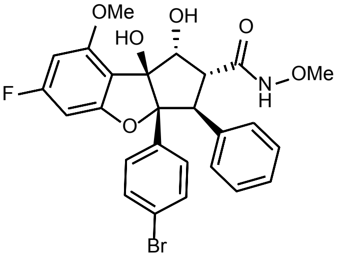	4.3	55.6
CMLD010515	(-)-RHT, rohitinib	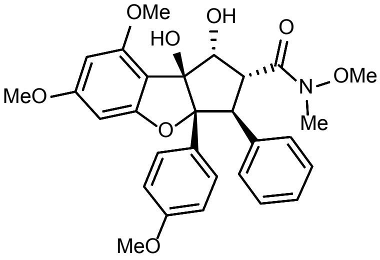	5.6	28.2
NSC326408	ROC, rocaglamide	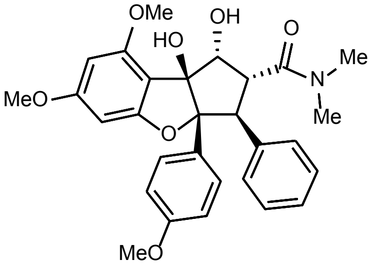	28.5	102
CMLD010512 (racemic)		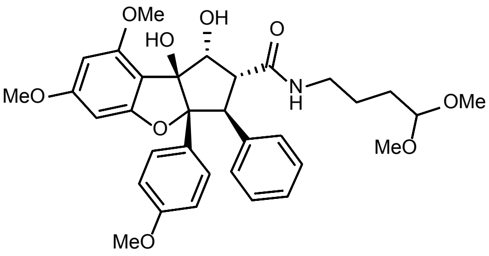	45.9	210

**S1.1 Amides and Hydroxamates**			*IC_{50} (nM)*	
Designation (used in pub)	*Other Name(s)*	*Structure*	*TRAIL*	*protein synthesis*
CMLD005522 (racemic)	CR-1-31B	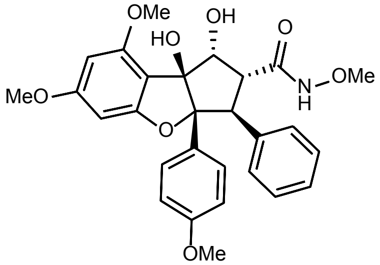	66.0	200
CMLD010428 (racemic)		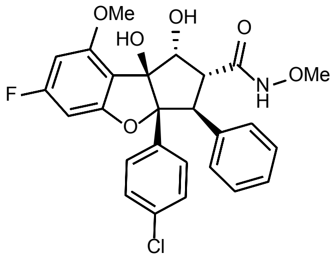	72.7	410
NSC705956	didesmethyl rocaglamide	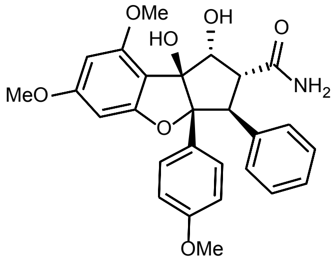	134	248
CMLD010331 (racemic)		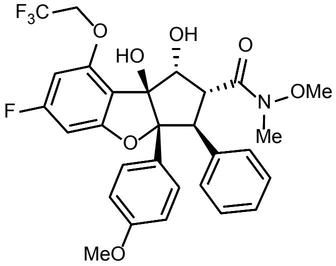	204	1403
CMLD010514 (racemic)		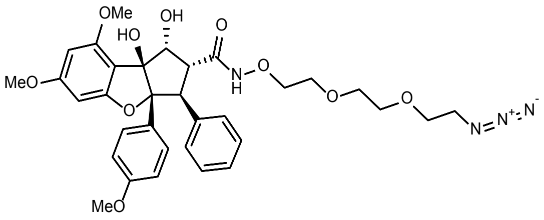	365	1233
CMLD010423 (racemic)		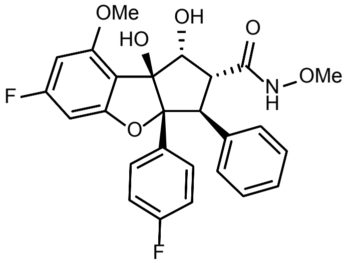	368	7180

**S1.2 Ketones**			*IC_{50} (nM)*	
Designation	*Other Name(s)*	*Structure*	*TRAIL*	*protein synthesis*
CMLD010536	(-)-furanyl rocaglate	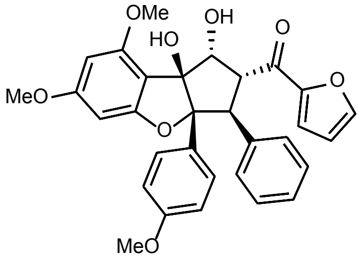	4.5	15.3
CMLD005551 (racemic)		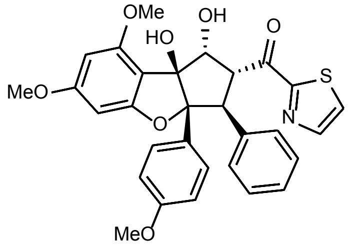	871	437
CMLD005557 (racemic)		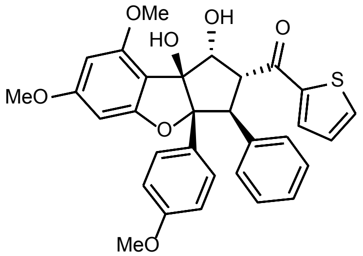	>1000	>1000

**S1.3 Oxazinones and pyridazones**			*IC_{50} (nM)*	
Designation	*Other Name(s)*	*Structure*	*TRAIL*	*protein synthesis*
CMLD010535 (racemic)	(±)-oxazinone	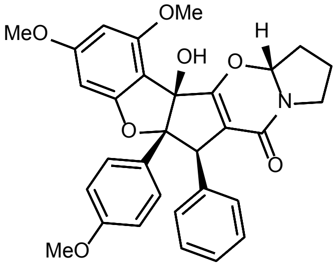	16.9	18.5
CMLD010506	(-)-aglaroxin C	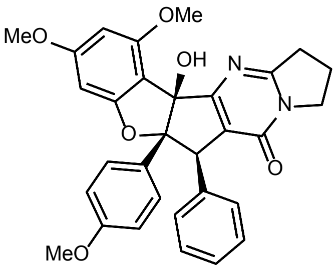	54.5	63.7

**S1.3 Oxazinones and pyridazones**			*IC_{50} (nM)*	
Designation	*Other Name(s)*	*Structure*	*TRAIL*	*protein synthesis*
CMLD010483 (racemic)		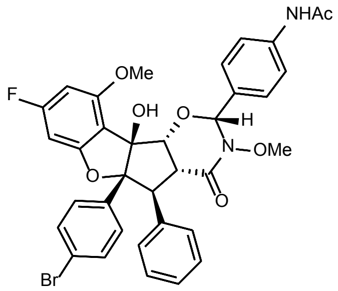	110	85.9
CMLD010582 (racemic)		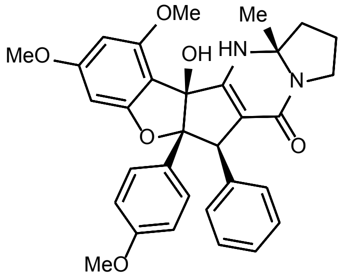	383	2172
CMLD010484 (racemic)		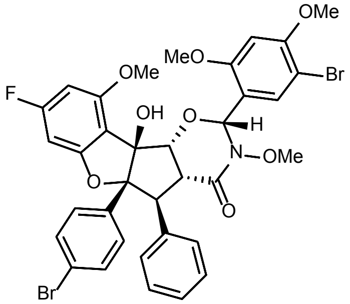	>1000	>1000

**S1.4 Rocaglaols**			*IC_{50} (nM)*	
Designation	*Other Name(s)*	*Structure*	*TRAIL*	*protein synthesis*
CMLD010517 (racemic)	FL3	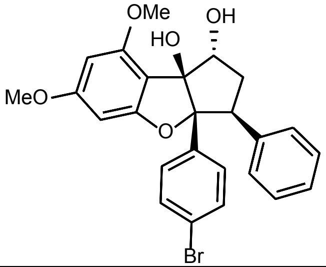	22.9	49.7
CMLD010518 (racemic)	(±)-rocaglaol	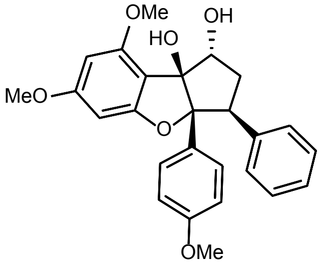	43.0	283

**S1.4 Rocaglaols**			*IC_{50} (nM)*	
Designation	*Other Name(s)*	*Structure*	*TRAIL*	*protein synthesis*
CMLD010585	(+)-rocaglaol	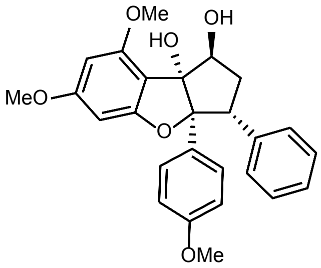	>1000	>1000
CMLD010586	(+)-rocaglaol ketone	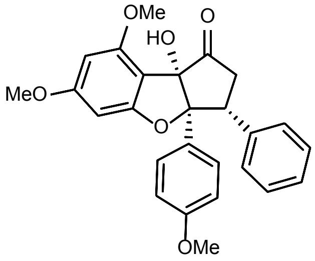	>1000	>1000

**S1.5 Esters**			*IC_{50} (nM)*	
Designation	*Other Name(s)*	*Structure*	*TRAIL*	*protein synthesis*
CMLD010337 (racemic)		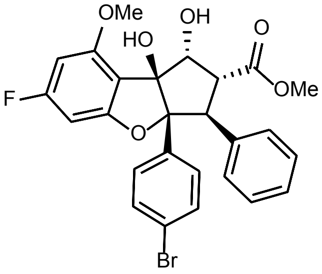	79.9	65.1
CMLD010516 (racemic)		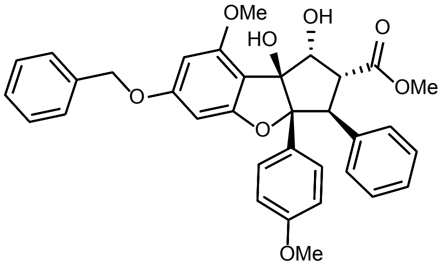	399	5701
CMLD010852 (racemic)		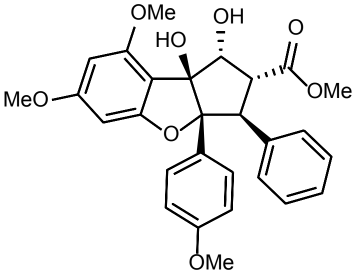	662	1043

S1.5 Esters			*IC*	
Designation	*Other Name(s)*	*Structure*	*TRAIL*	*protein synthesis*
CMLD000069 (racemic)		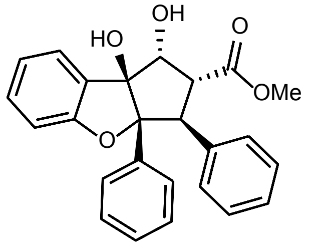	>1000	>1000
CMLD007564 (racemic)		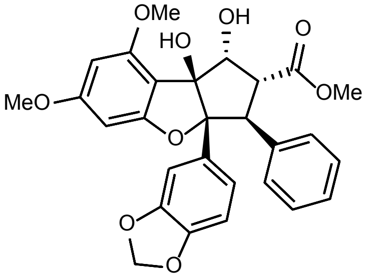	>1000	>1000
CMLD007565 (racemic)		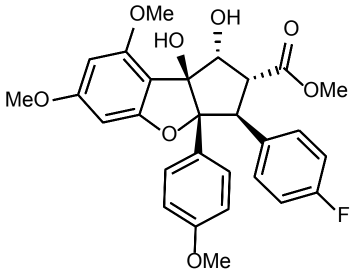	>1000	>1000
CMLD010507		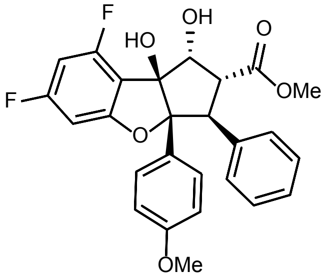	>1000	>1000
CMLD010534	silvestrol dibenzyl ether	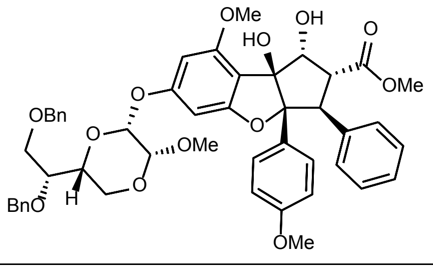	>1000	>1000

**S1.6 Miscellaneous structure classes (sorted by activity)**			*IC_{50} (nM)*	
Designation	*Other Name(s)*	*Structure*	*TRAIL*	*protein synthesis*
CMLD010338 (racemic)		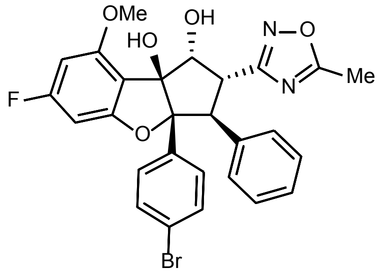	30.1	61.0
CMLD010343 (racemic)		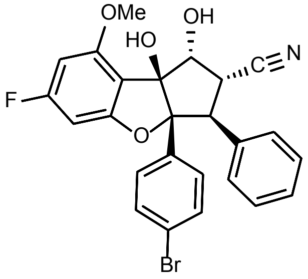	32.2	95.8
CMLD010336 (racemic)		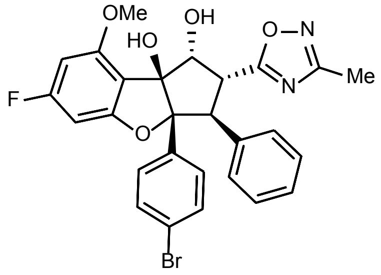	36.1	78.4
CMLD010332 (racemic)		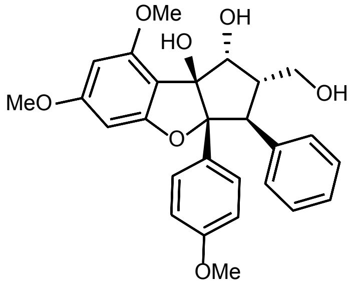	592	1369
CMLD010425 (racemic)		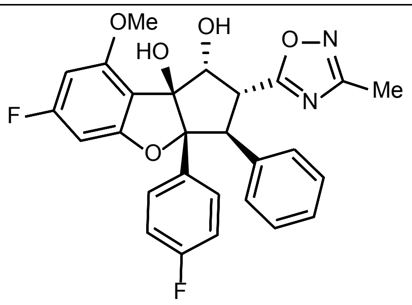	770	>1000

**S1.6 Miscellaneous structure classes (sorted by activity)**			*IC_{50} (nM)*	
Designation	*Other Name(s)*	*Structure*	*TRAIL*	*protein synthesis*
CMLD010426 (racemic)		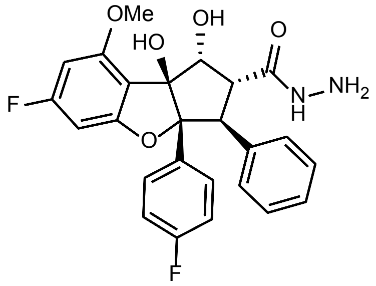	>1000	>1000

**S1.7 "Exo" rocaglates**			*IC_{50} (nM)*	
Designation	*Other Name(s)*	*Structure*	*TRAIL*	*protein synthesis*
CMLD010430 (racemic)	exo-CMLD010337	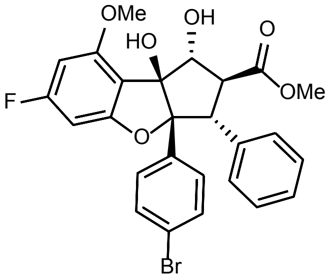	543	1998
CMLD010442 (racemic)	exo-CR-1-31B	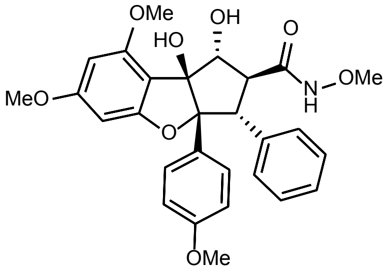	679	1900
CMLD010441 (racemic)	exo-RHT	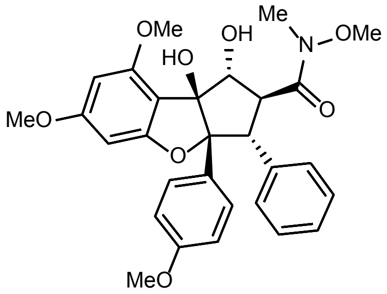	>1000	>1000

**S1.7 "Exo" rocaglates**			*IC_{50} (nM)*	
Designation	*Other Name(s)*	*Structure*	*TRAIL*	*protein synthesis*
CMLD010583 (racemic)	exo-CMLD010343	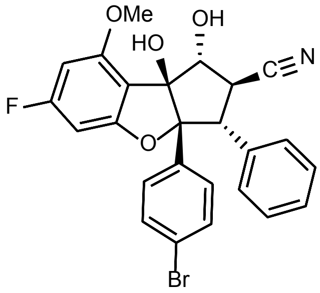	>1000	>1000
CMLD010584 (racemic)		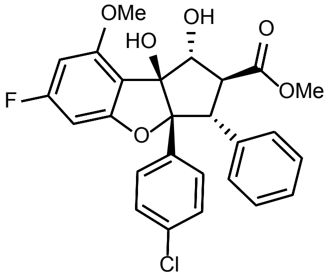	>1000	>1000
CMLD010429 (racemic)	exo-methyl rocaglate	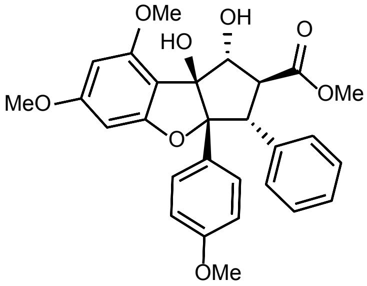	>1000	>1000

Aglains, rocaglate lactones, and related scaffolds
(all inactive, *i.e.* IC_{50} > 1000 nM for both TRAIL activation and protein synthesis inhibition)

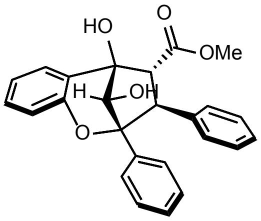

DCIII-92

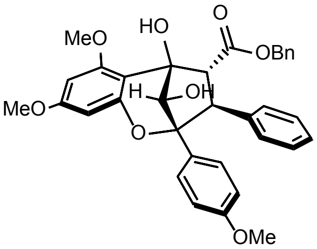

CMLD008820

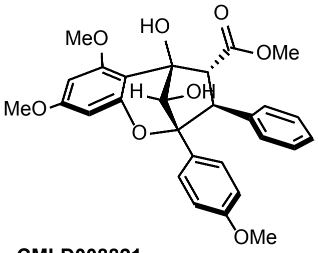

CMLD008821 (racemic)
CMLD010499 (96% ee)
CMLD010498 (99% ee)
CMLD010533 (mixture of alcohol diasteromers)

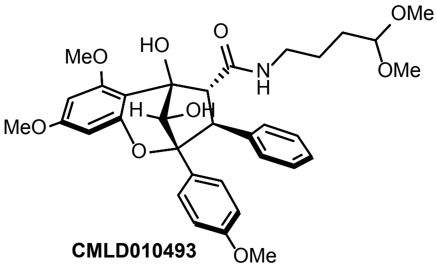

CMLD010493

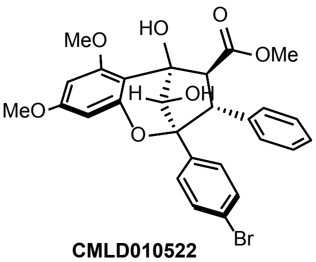

CMLD010522

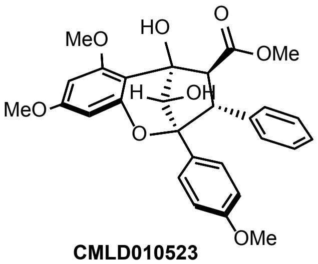

CMLD010523

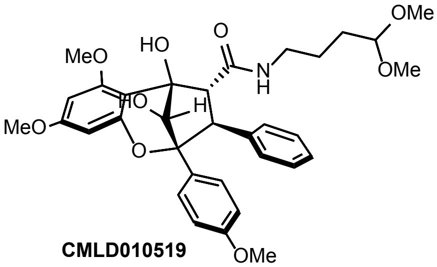

CMLD010519

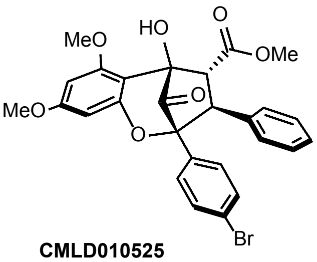

CMLD010525

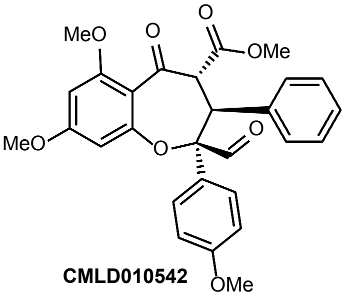

CMLD010542

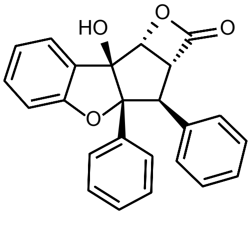

CMLD010360
CMLD010528

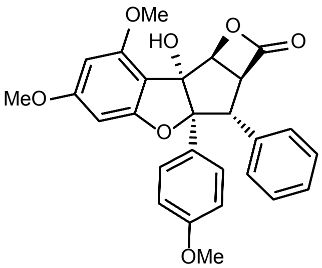

CMLD010527

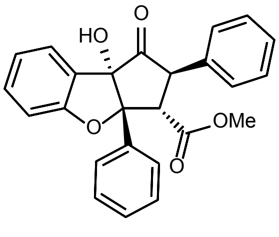

CMLD000096
(racemic)

SUPPLEMENTARY FIGURE S1: *Effects of rocaglates on ACHN renal carcinoma cell growth/survival*:

ACHN cells were pre-treated for 4 h in the presence of variable concentrations of rocaglates followed by 18-24 h in the presence or absence of TRAIL (40 ng/ml final). Cell numbers were assessed using the XTT assay and normalized to untreated control cells on the same assay plate. Error bars (n = 2-6) not included for clarity.

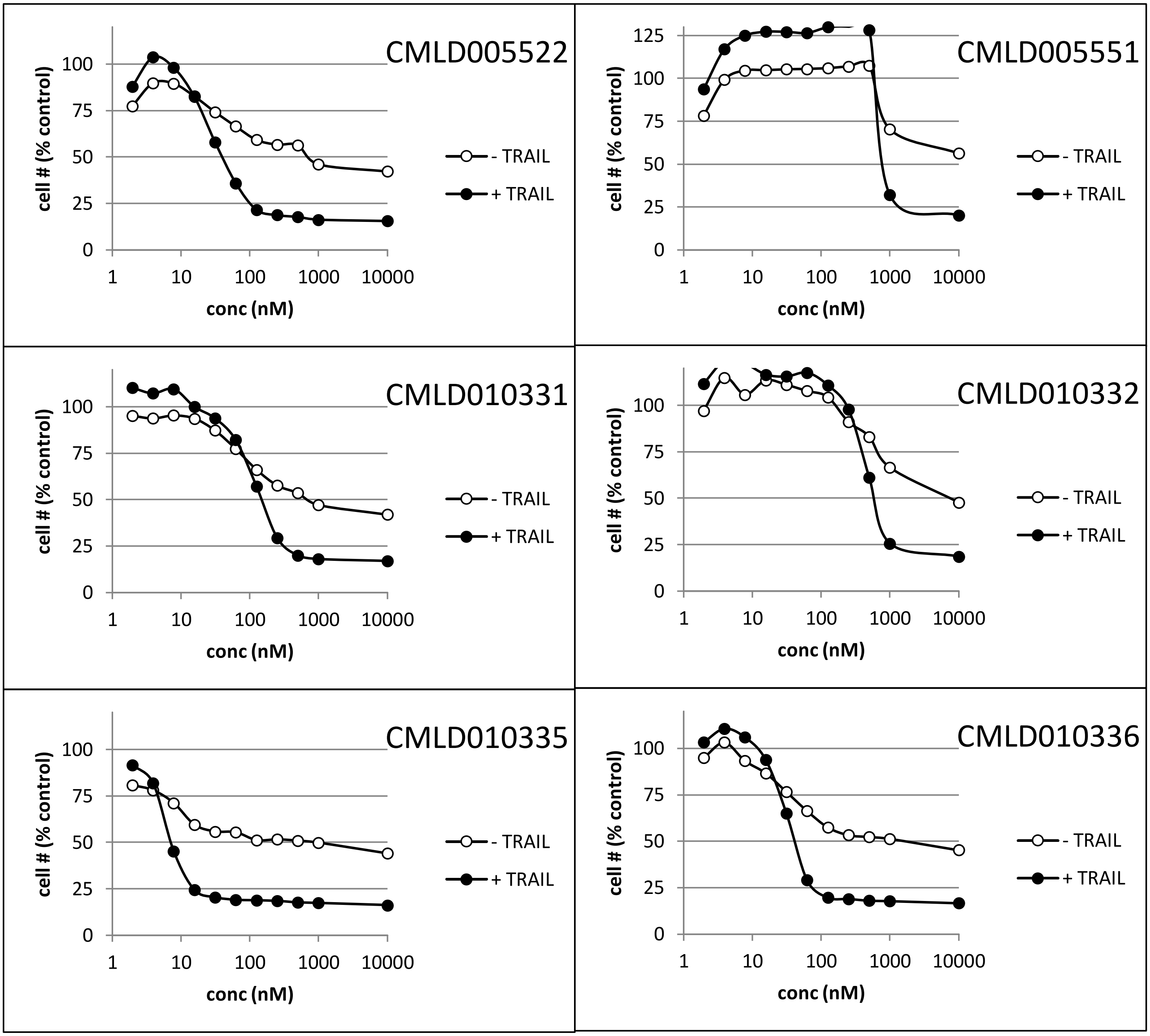

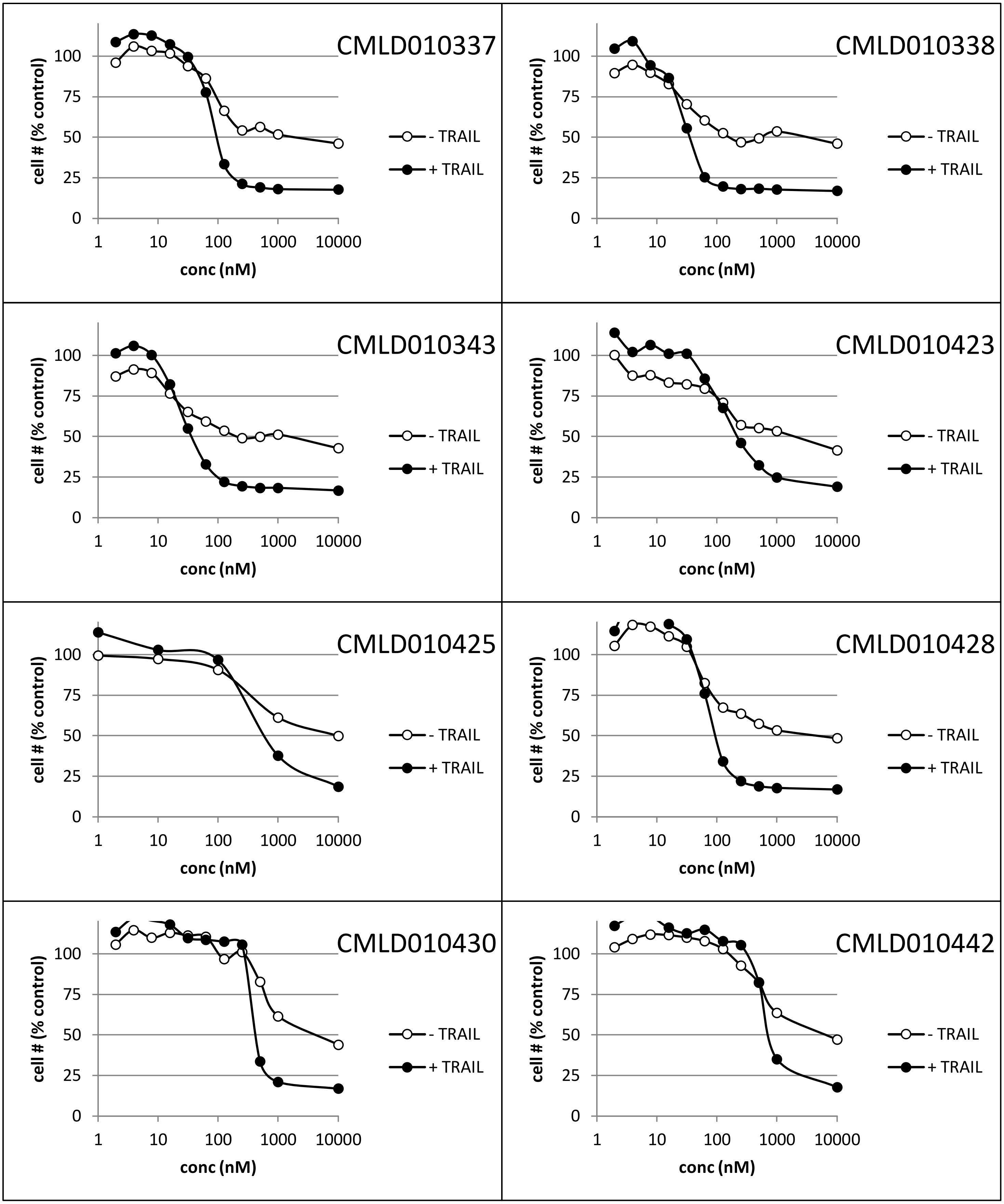
CMLD010337
CMLD010338
CMLD010343
CMLD010423
CMLD010425
CMLD010428
CMLD010430
CMLD010442
cell # (% control)
0
25
50
75
100
conc (nM)
1
10
100
1000
10000
- TRAIL
+ TRAIL

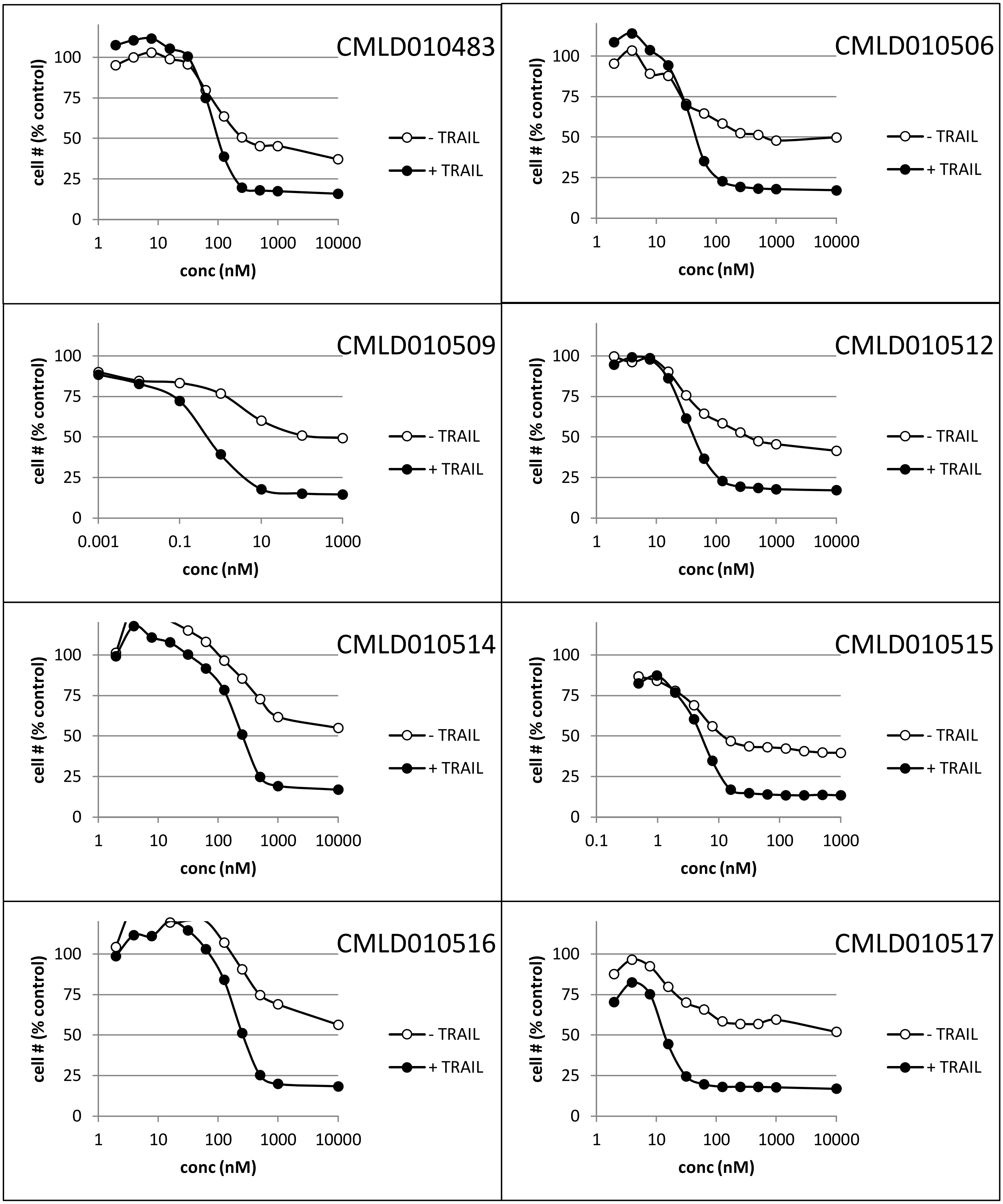
CMLD010483
CMLD010506
CMLD010509
CMLD010512
CMLD010514
CMLD010515
CMLD010516
CMLD010517
cell # (% control)
conc (nM)
- TRAIL
+ TRAIL
0
25
50
75
100
0.001
0.1
1
10
100
1000
10000

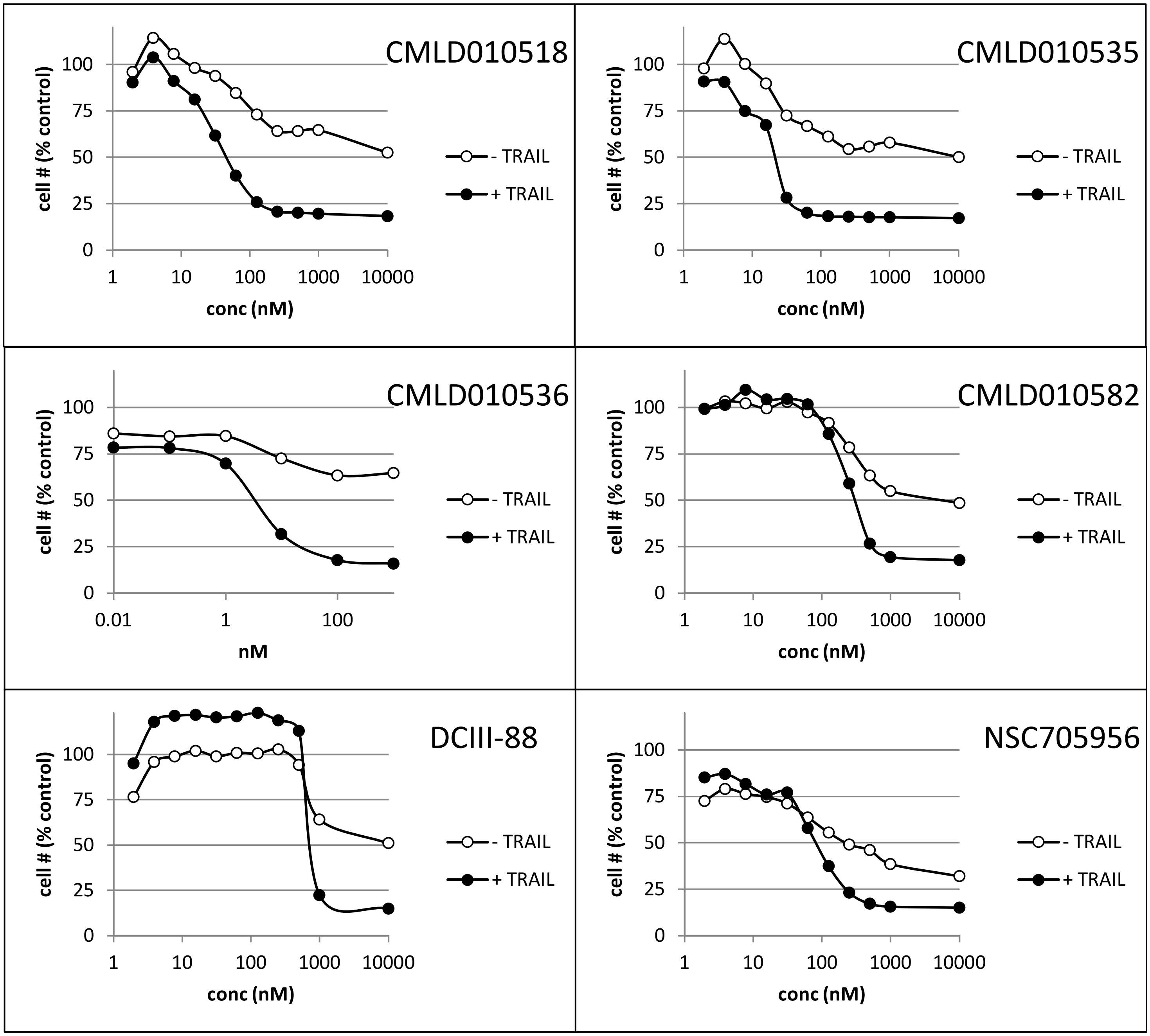
CMLD010518
cell # (% control)
conc (nM)
- TRAIL
+ TRAIL
0
25
50
75
100
1
10
100
1000
10000
CMLD010535
cell # (% control)
conc (nM)
- TRAIL
+ TRAIL
CMLD010536
cell # (% control)
nM
0.01
1
100
- TRAIL
+ TRAIL
CMLD010582
cell # (% control)
conc (nM)
- TRAIL
+ TRAIL
DCIII-88
cell # (% control)
conc (nM)
- TRAIL
+ TRAIL
NSC705956
cell # (% control)
conc (nM)
- TRAIL
+ TRAIL

SUPPLEMENTARY FIGURE S2: *Effects of the 4 most potent rocaglates on death receptor signaling*:

A: Cells were treated for 4 h with the indicated rocaglate at 100 nM followed by 4 h TRAIL and assessment of caspase 8 activity or 12 h TRAIL and assessment of caspase 3 activity. Results were normalized to untreated (DMSO, - TRAIL) controls on the same plate. Error bars represent ± sd (n = 3).

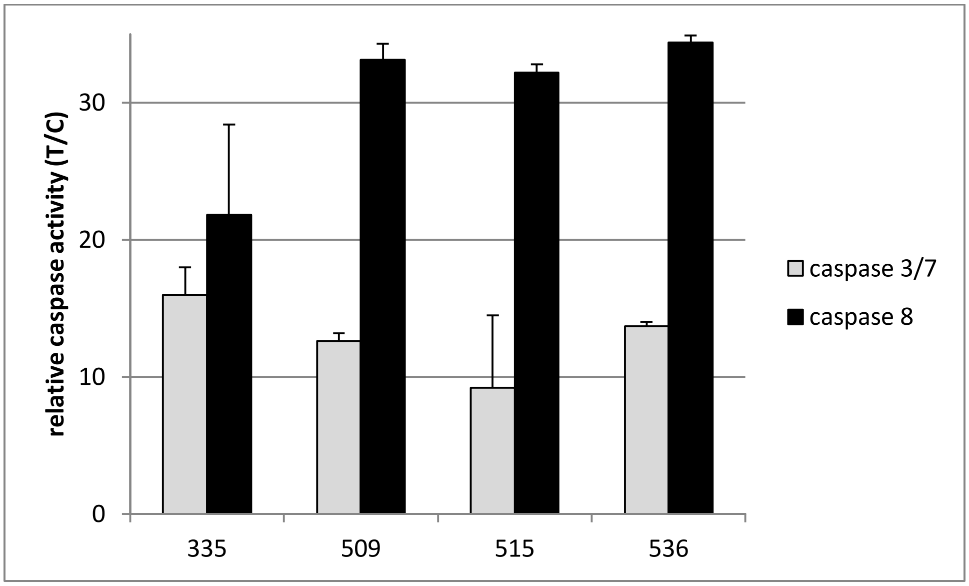

B: Cells were pretreated for 2 h with or without 100 µM Z-VADFMK followed by 4 h ± rocaglate (100 nM final concentration), then ± TRAIL for 18 h and assessment of cell survival by XTT. Error bars represent ± sd (n = 4).

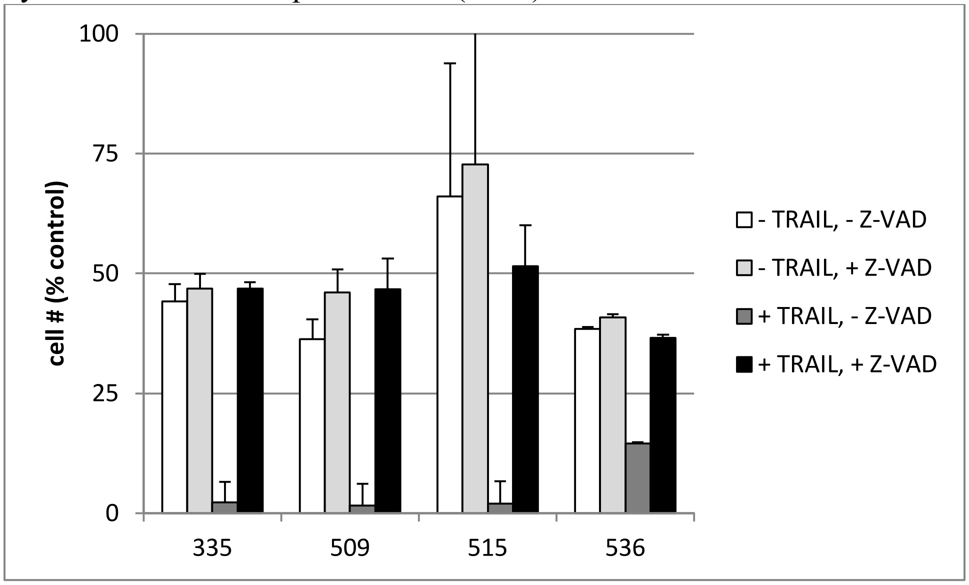

C: Caspase 3 activity was assessed after 72 h incubation with the indicated rocaglate (100 nM final concentration) and normalized to untreated control on the same plate. Error bars represent ± sd (n = 4).

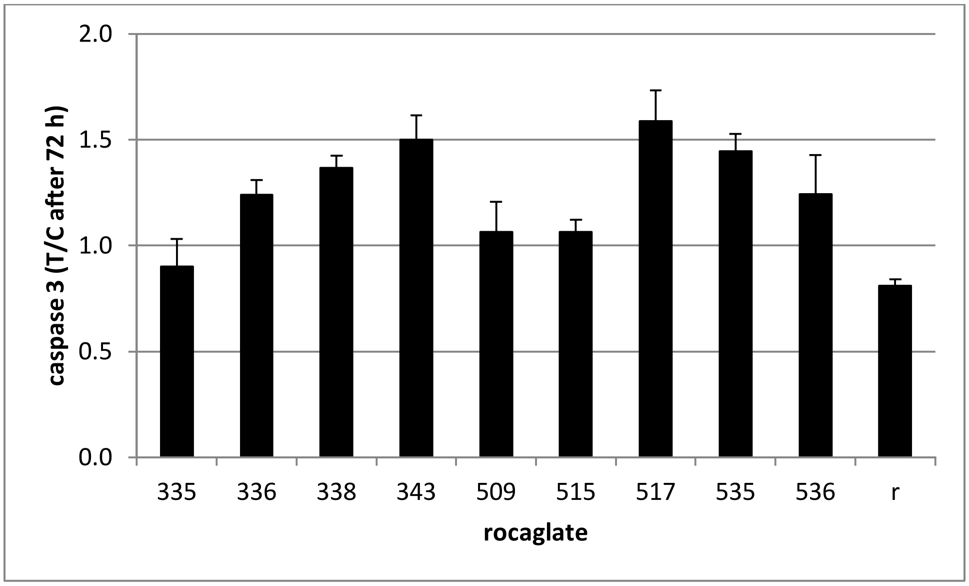

SUPPLEMENTARY FIGURE S3: *Effects of the rocaglates on cFLIP protein expression*:

Cells were treated for 4 h with the indicated rocaglates at 10, 100, or 1000 nM followed by quantitative western blot analysis of cFLIP isoforms. Values were normalized to loading control (GAPDH) and expressed as a % of signal for untreated cells run on the same gel. Results are sorted by IC_{50} from most to least potent for TRAIL sensitization (see Supplementary Table 1 for values).

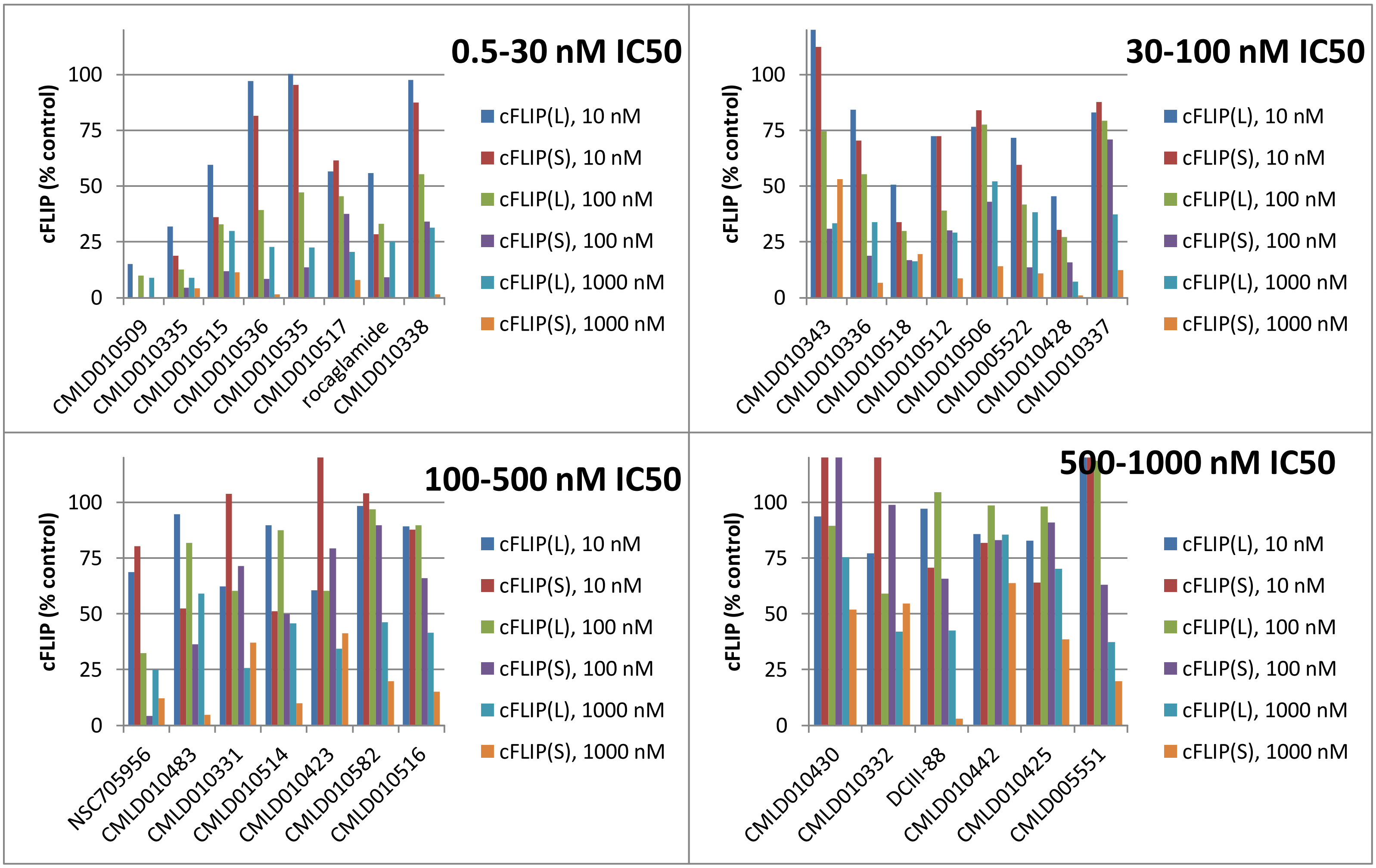

SUPPLEMENTARY FIGURE S4: *Rocaglate effects are unaffected by inhibition of MEK, JNK, or ROS* :

Cells were pretreated for 1 h with either the MEK inhibitor UO126 (20 µM), the JNK inhibitor SP600125 (50 µM), or N-acetyl cysteine (NAC, 10 mM) followed by the indicated rocaglate (1000 nM final) for 4 h, then ± TRAIL for 24 h. Cell survival was estimated using the XTT assay. However, NAC interferes with this assay, so for that experiment, cell survival was estimated by assessment of total protein using an SRB assay. Cell numbers were normalized to untreated (vehicle) control and reported as % of control values.

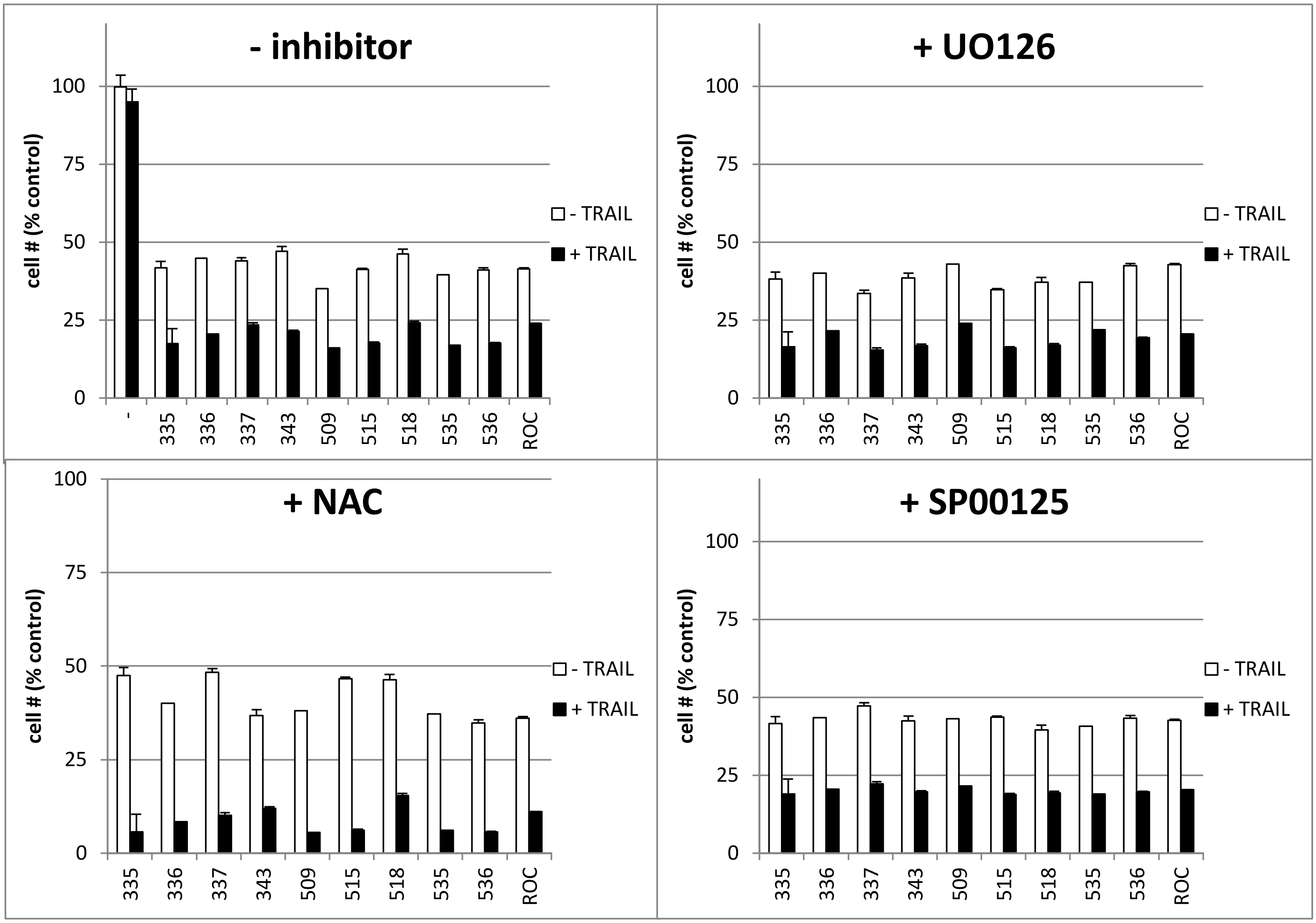

SUPPLEMENTARY FIGURE S5: *Rocaglamide effects on selected other proteins:* Cells were pretreated for 1-24 h with rocaglamide followed by western blot analysis.

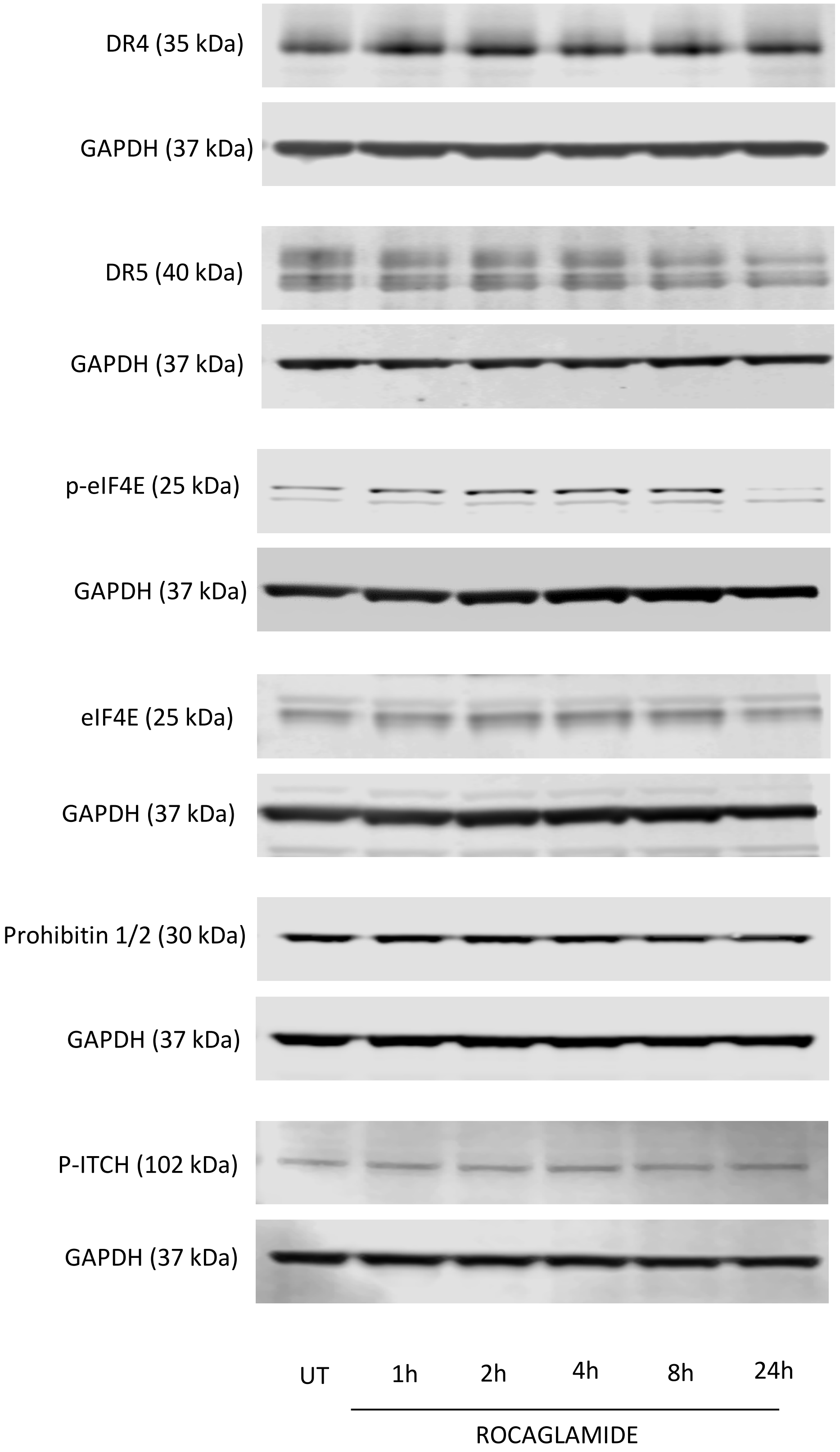

Uncropped blots (SUPPL FIG S5):
GREEN: rabbit primary antibody, detection with goat anti-rabbit (800)
RED: mouse primary antibody, detection with goat anti-mouse (680)
MW markers – RED NOTE: single blot, simultaneous probes, visualization in two channels

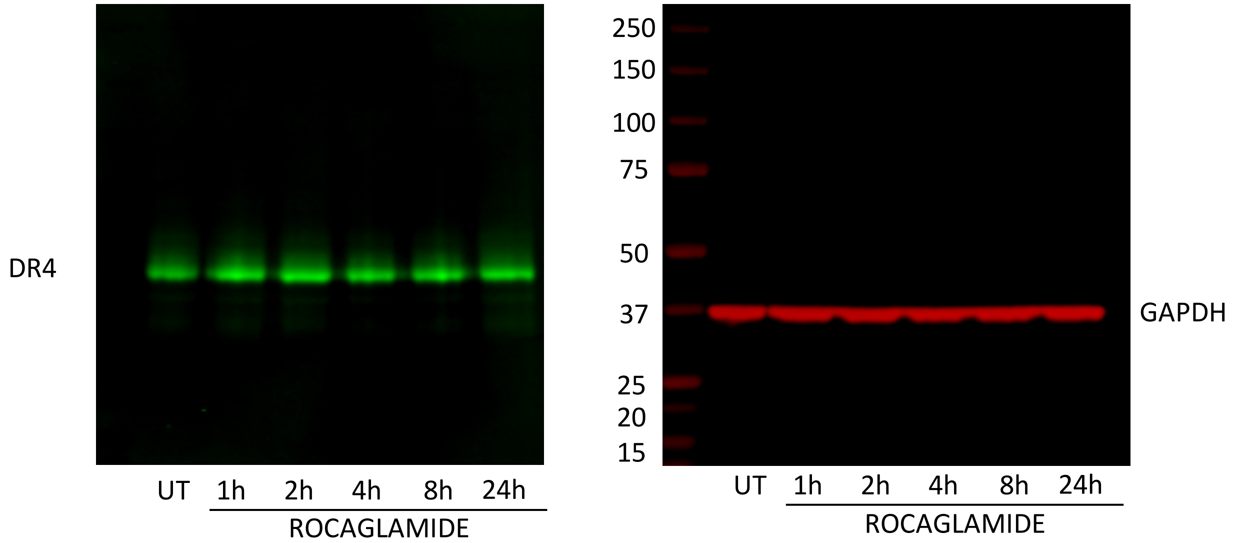

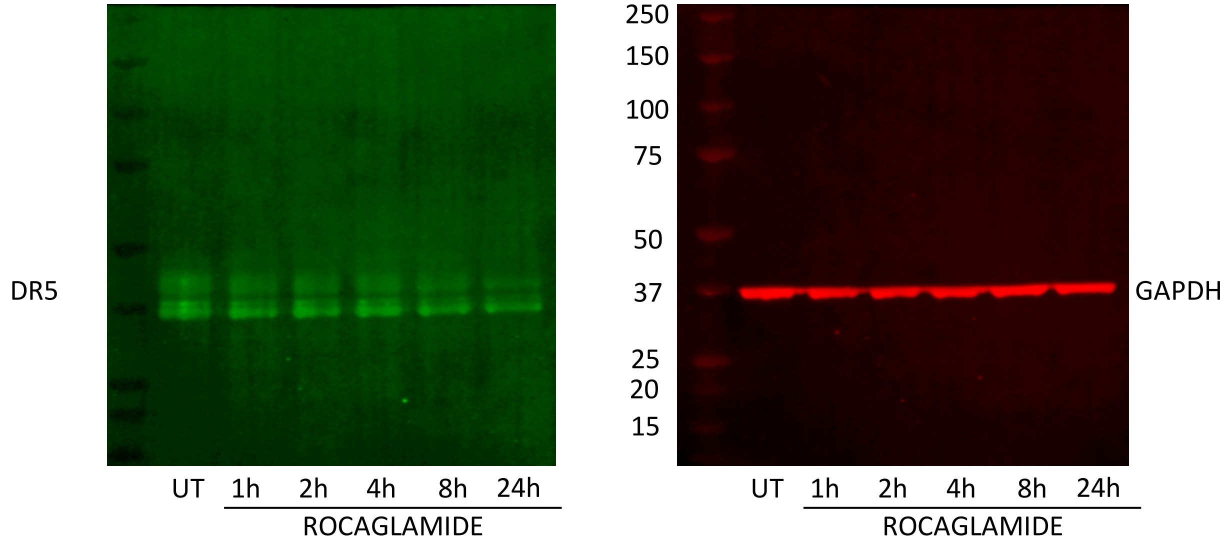

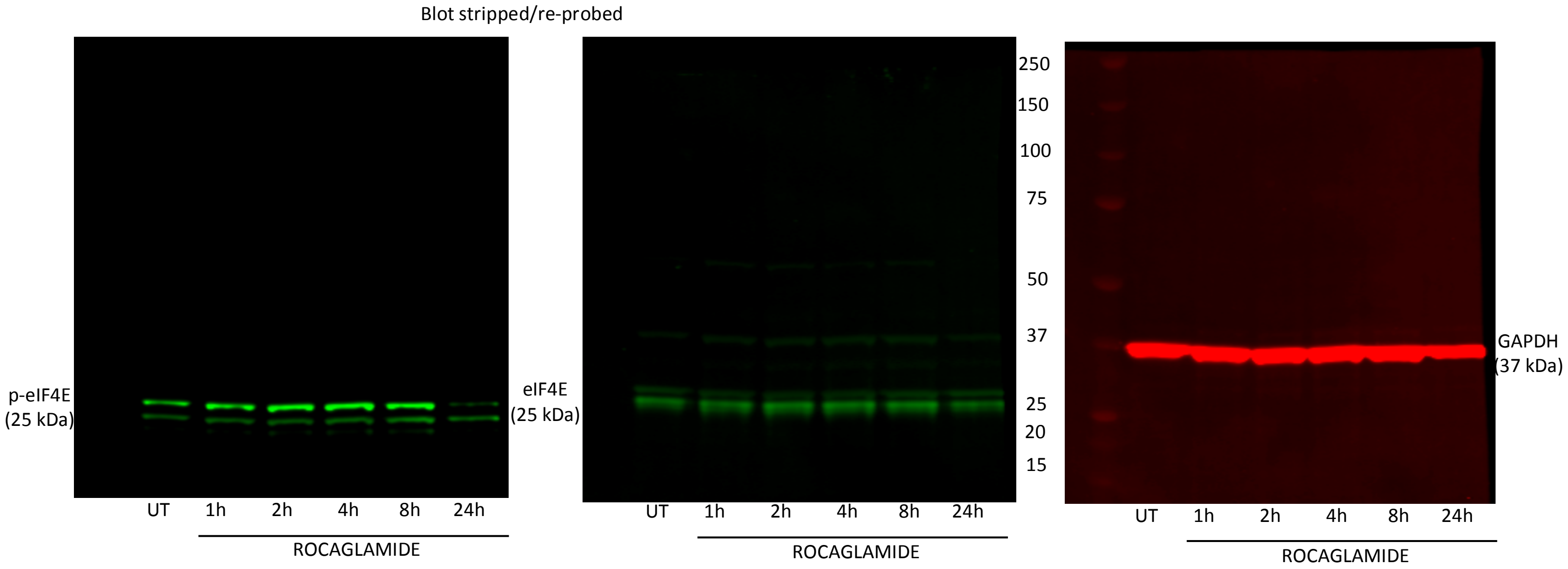

Uncropped blots (SUPPL FIG S5):
GREEN: rabbit primary antibody, detection with goat anti-rabbit (800)
RED: mouse primary antibody, detection with goat anti-mouse (680)
MW markers - RED NOTE: single blot, simultaneous probes, visualization in two channels

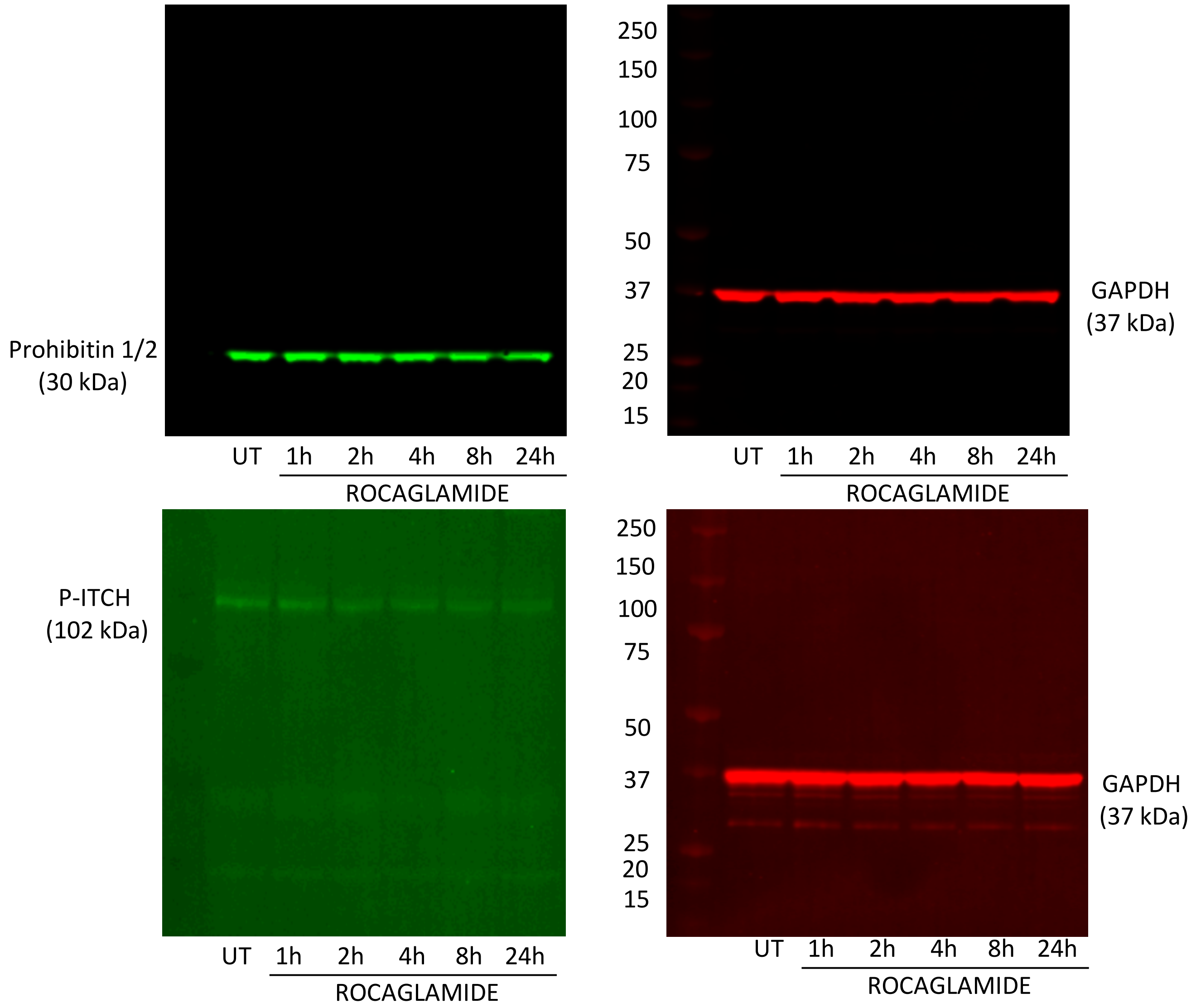

SUPPLEMENTARY FIGURE S6: *Effect of rocaglamide on surface expression of TRAIL receptors*:

ACHN cells were treated for 24 h with ROC followed by flow cytometric analysis of surface expression of TRAIL receptors DR4, DR5, and “decoy” receptors TR3 (DcR1) and TR4 (DcR2).

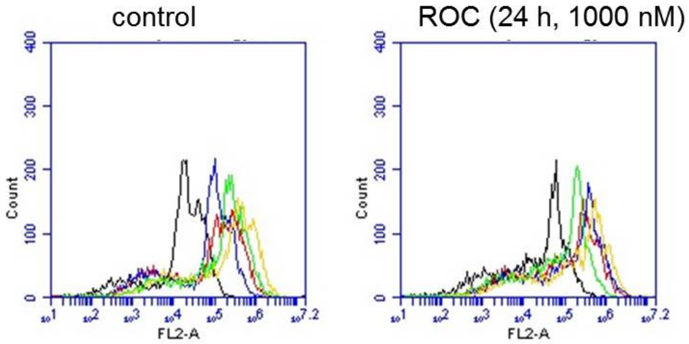

Black: isotype control
Red: DR4
Blue: DR5
Orange: TR3 (DcR1)
Green: TR4 (DcR2)

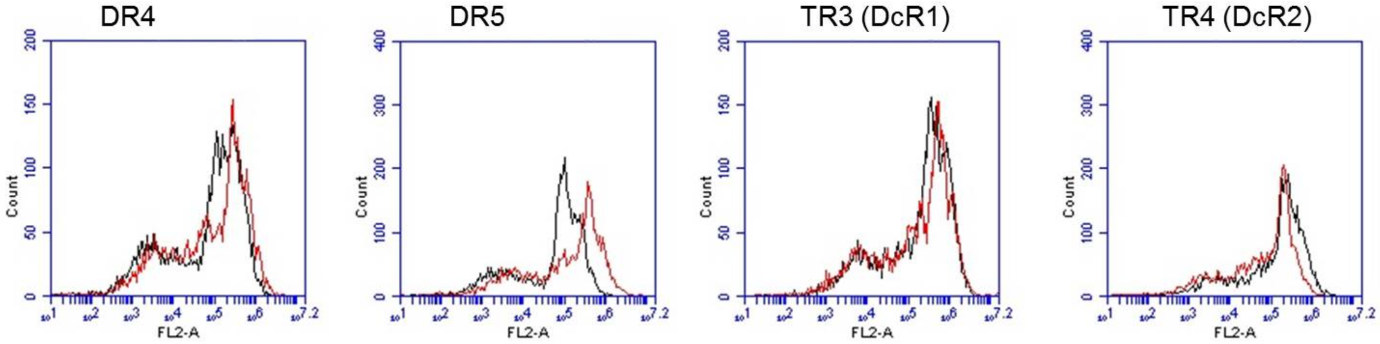

Black: vehicle control
Red: ROC-treated

Top panel shows vehicle control cells (left) and ROC-treated cells (right) while the bottom panel shows vehicle control vs. ROC treated cells for each receptor.

PE-conjugated antibodies were obtained from ThermoFisher and used according to the manufacturers recommendations.
Antibodies: DR4 (clone DJR1), DR5 (clone DJR2-4), TR3 (clone DJR3), TR4 (clone TRAIL-R4-01)
Data were acquired on an Accuri C6 flow cytometer and analyzed using the instrument’s CFLOW software (Accuri Cytometers, Ann Arbor, MI, USA).

SUPPLEMENTARY FIGURE S7: *Effect of rocaglamide and analogs on CAKI-1 and SN12C RCC cells*:

CAKI-1 and SN12C cells were pre-treated for 4 h in the presence of variable concentrations of rocaglates followed by 18-24 h in the presence or absence of TRAIL (40 ng/ml final). Cell numbers were assessed using the XTT assay and normalized to untreated control cells on the same assay plate (left panels below). In parallel, cells were treated for 4 h with rocaglates followed by analysis of protein synthesis (right panels below). Error bars represent sd, n = 3-4.

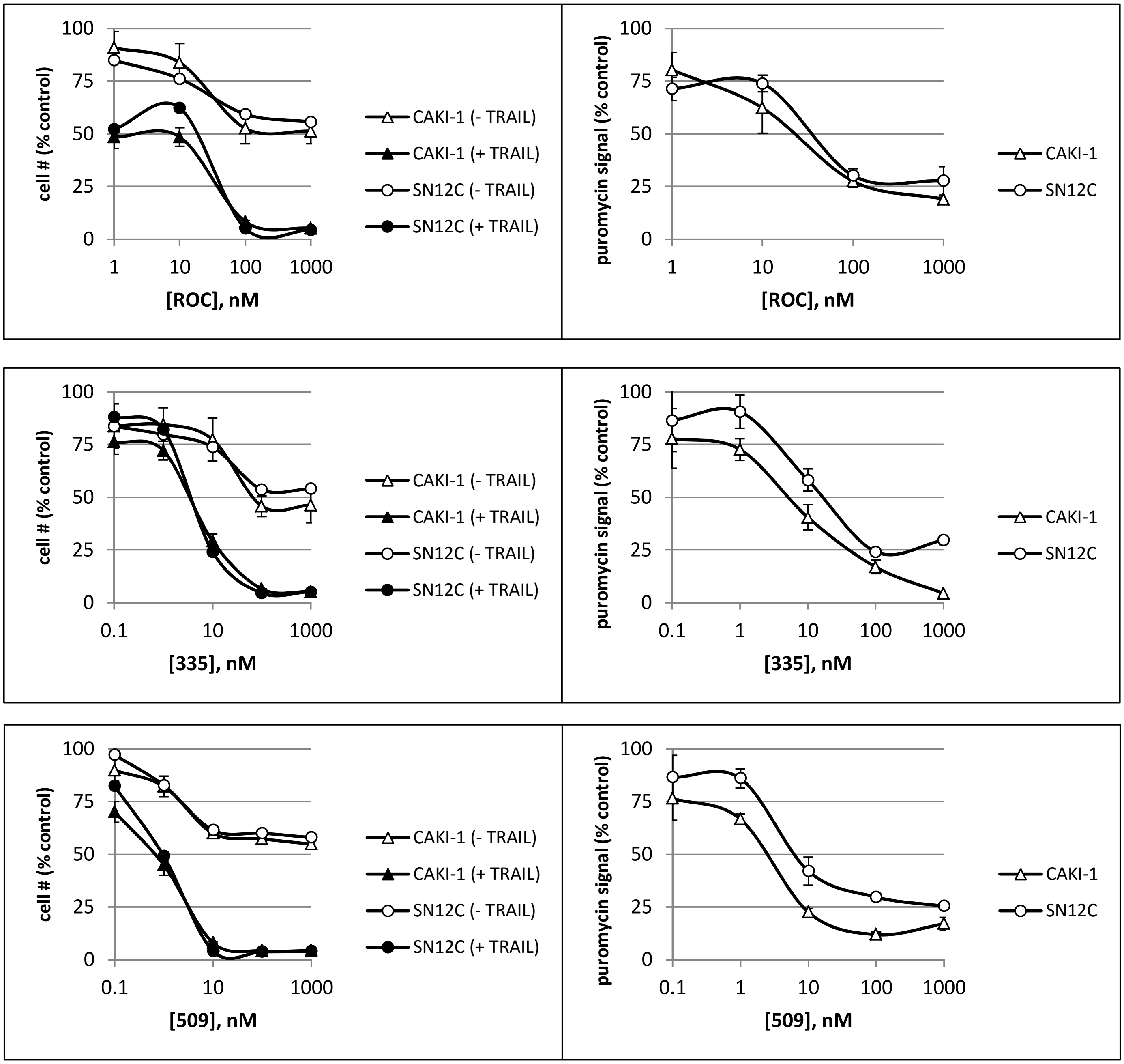

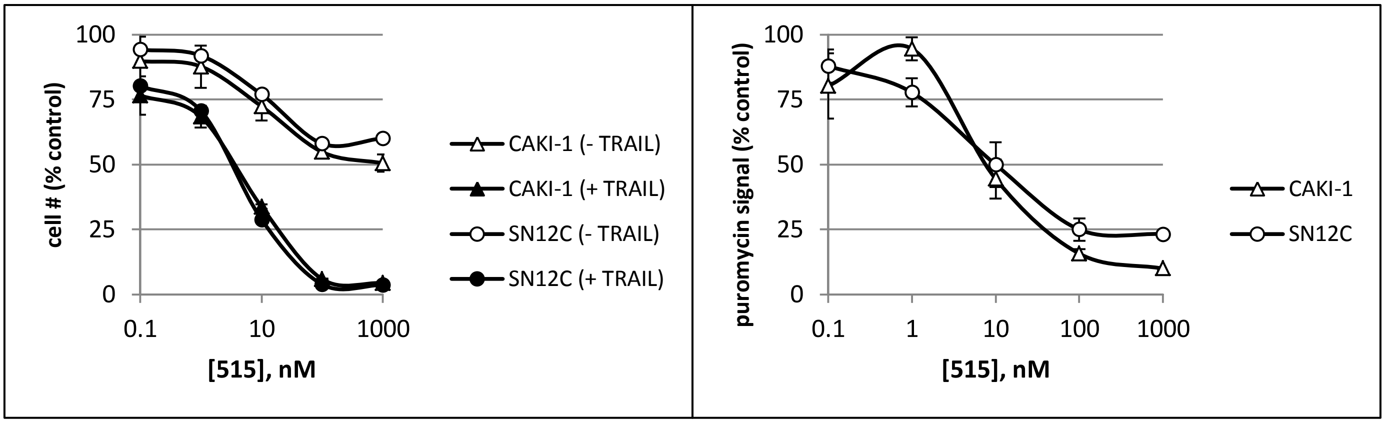

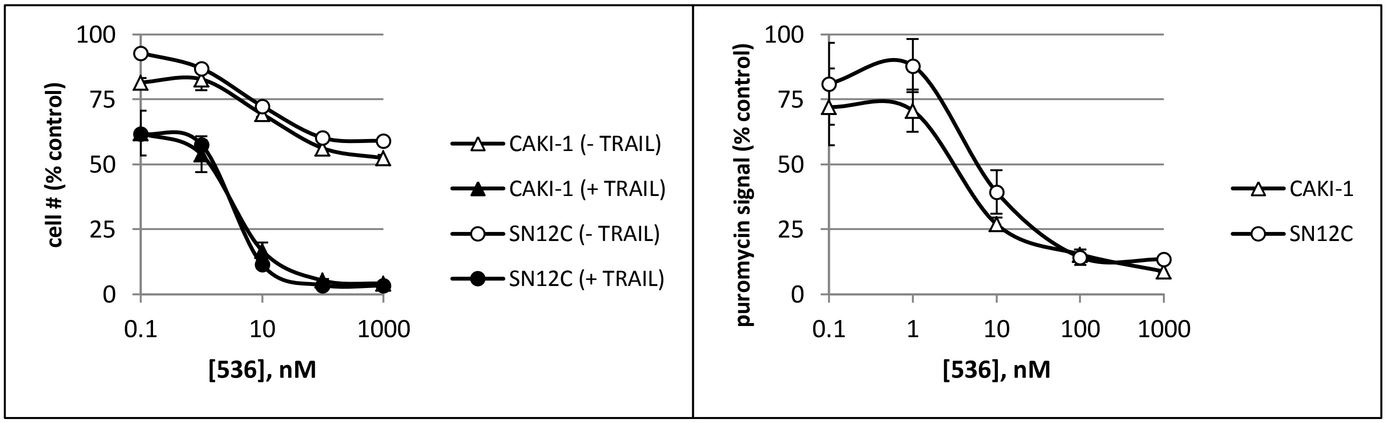

IC_{50} (nM) values derived from above data

Compound	CAKI (+ TRAIL)	CAKI (ptn synth)	SN12C (+ TRAIL)	SN12C (ptn synth)
**ROC**	13.3	20.1	6.9	76.0
**335**	6.8	10.0	4.9	10.0
**509**	1.3	2.9	1.1	6.1
**515**	7.1	9.7	5.6	6.8
**536**	3.8	5.7	3.6	9.4

SUPPLEMENTARY FIGURE S8: *Effect of rocaglamide and analogs on human renal epithelial cells*:

HRE cells were pre-treated for 4 h in the presence of variable concentrations of rocaglates followed by 18-24 h in the presence or absence of TRAIL (40 ng/ml final). Cell numbers were assessed using the XTT assay and normalized to untreated control cells on the same assay plate. In parallel, cells were treated for 4 h with rocaglates followed by analysis of protein synthesis (combined in last graph below).
Error bars represent sd, n = 4. Vertical bar represents ~IC_{50} value for sensitization of ACHN cells.

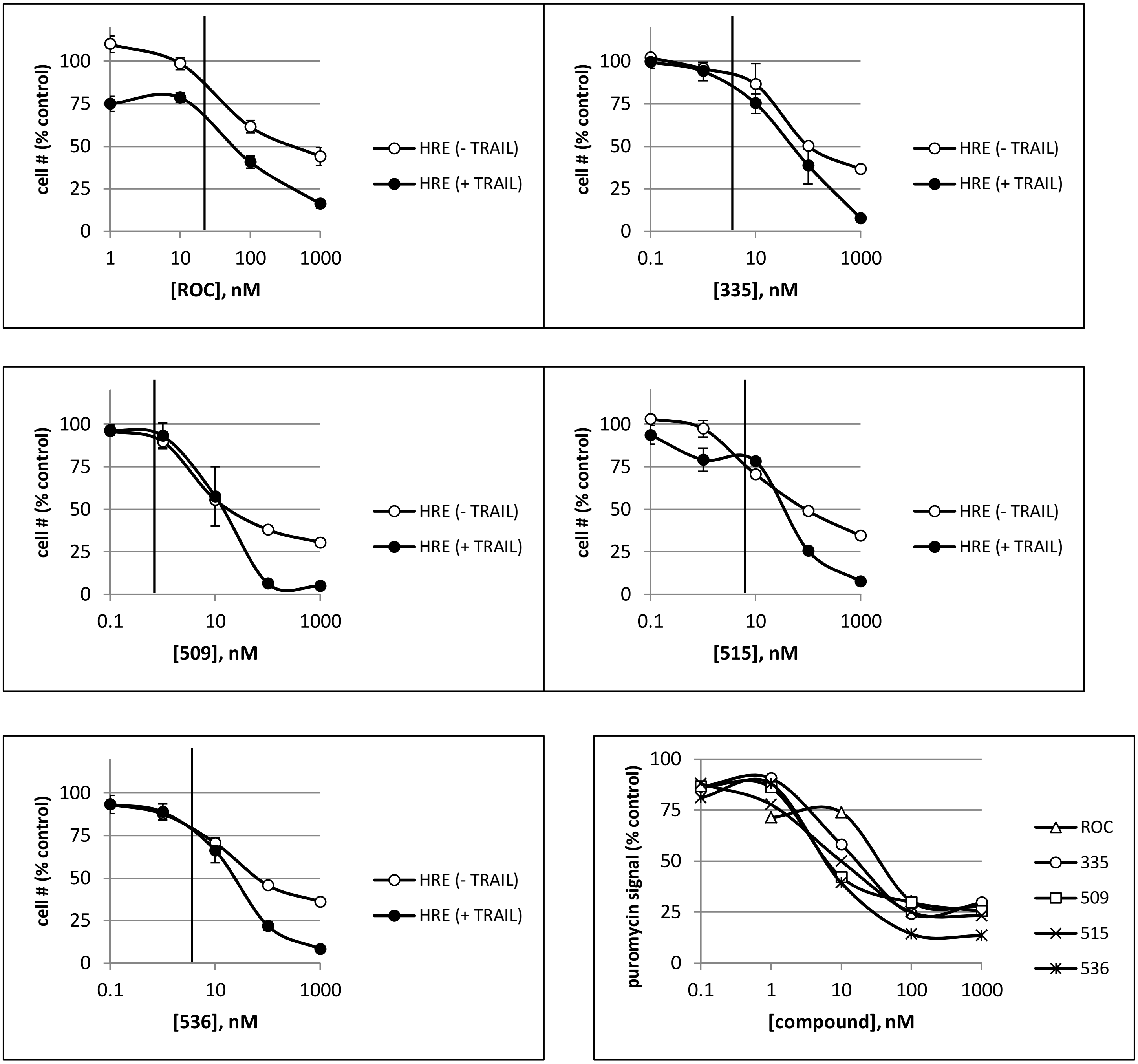

SUPPLEMENTARY FIGURE S9: *Rocaglamide does not affect expression of cFLIP mRNA:*

ACHN cells were pretreated for 4 h or 8h with 1 μM rocaglamide followed by total RNA extraction using Qiagen RNeasy Mini Kit. 2 μg of RNA was used to make cDNA using ThermoFisher High-Capacity reverse transcription kit in a 25 μL reaction. 5 μL of cDNA was used for a 50 μL PCR reaction using Qiagen PCR Master Mix kit. PCR conditions were 95 degrees 10 min, 95 degrees 45 sec, 55 degrees 45 sec, 72 degrees 45 sec for 25 cycles followed by a final extension at 72 degrees for 7 min. The following custom primers from IDT were used for detection and quantification of mRNA for cFLIP short and long form:

cFLIPL_Forward	GGA CCT TGT GGT TGA GTT GG
cFLIPL_Reverse	ATC AGG ACA ATG GGC ATA GG
cFLIPS_Forward	GGC TCC CAG AGT GTG TAT GG
cFLIPS_Reverse	AGC TTC TCG GTG AAC TGT GC

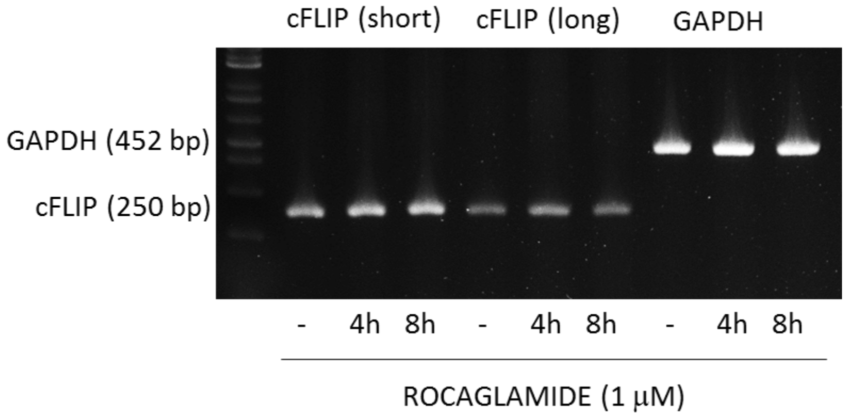

SUPPLEMENTARY FIGURE S10: *Original uncropped blots – data for Figures 3, 4, and 6.*

For all original uncropped blots,
GREEN: rabbit primary antibody, detection with goat anti-rabbit (800) secondary
RED: mouse primary antibody, detection with goat anti-mouse (680) secondary
Analysis on LiCor Odyssey – simultaneous analysis of red and green signals. For clarity, each individual signal is shown separately (side-by-side for same blot). In some cases 3 blots appear because the blot was stripped and re-probed.

NOTE: unlabeled lanes include extracts from cell extracts irrelevant to this manuscript.

EFFECT OF ROCAGLAMIDE ON cFLIP PROTEIN (main text Figure 3C).

4 h rocaglamide

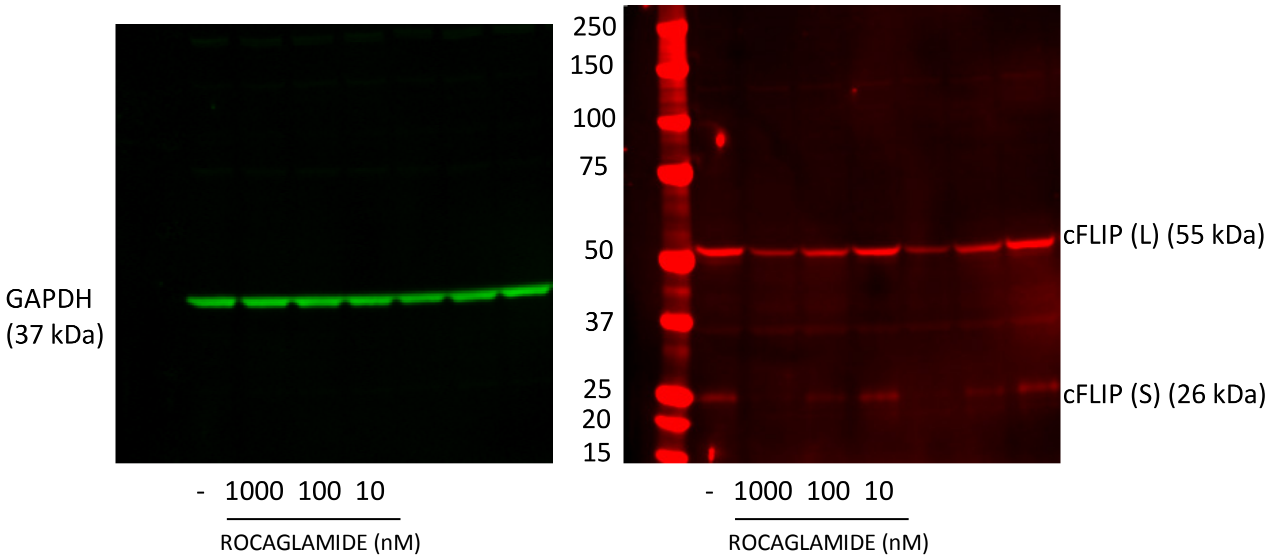

24 h rocaglamide

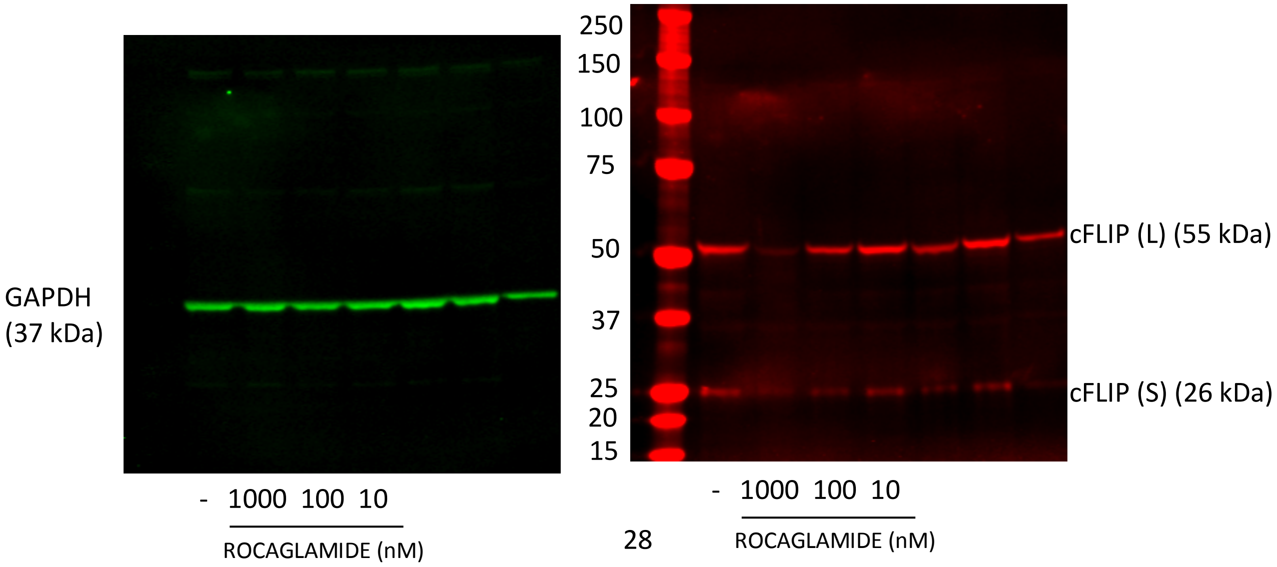

EFFECTS OF ROCAGLAMIDE ON MITOCHONDRIAL PROTEINS (main text Fig. 4A)

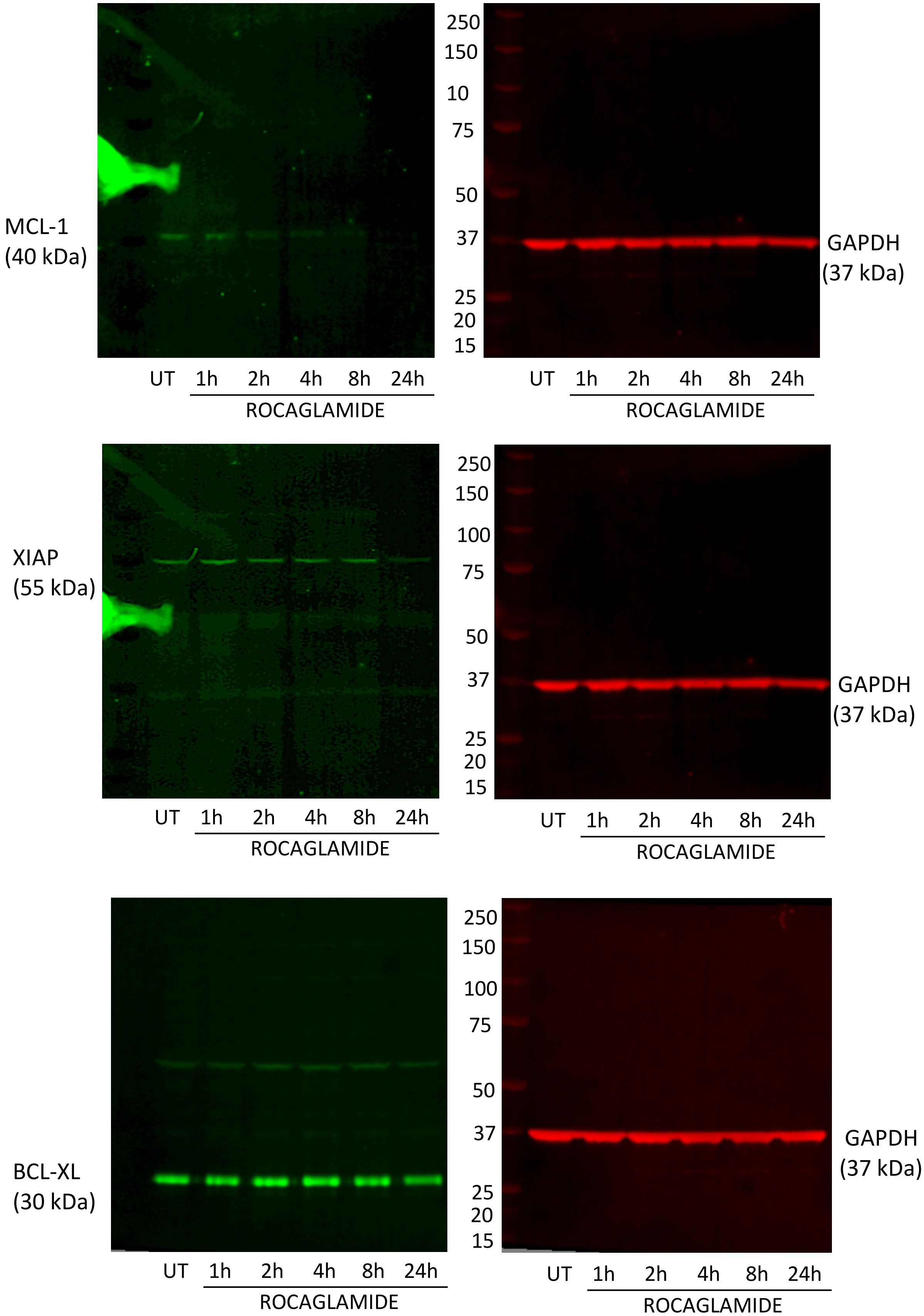

EFFECTS OF ROCAGLAMIDE ON MITOCHONDRIAL PROTEINS (main text Fig. 4A)

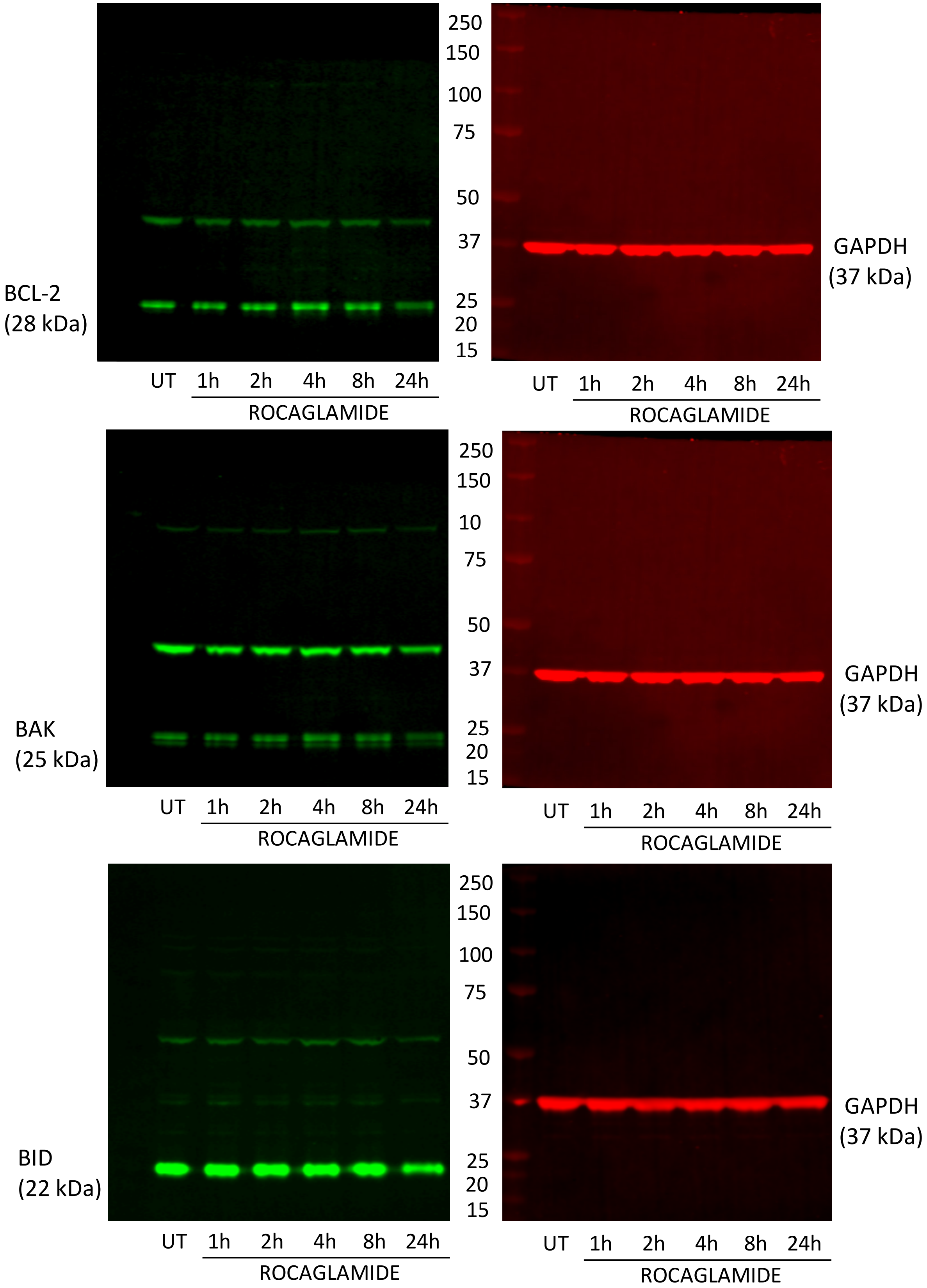

EFFECTS OF ROCAGLAMIDE ON MITOCHONDRIAL PROTEINS (main text Fig. 4A)

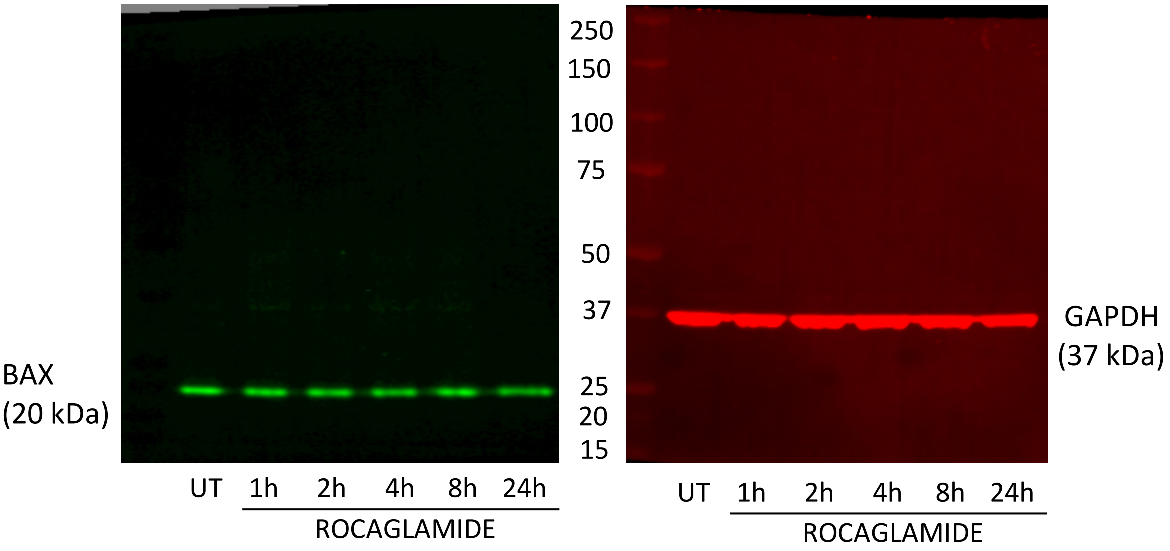

EFFECT OF ROCAGLATES ON MCL-1 (main text Fig 4C)

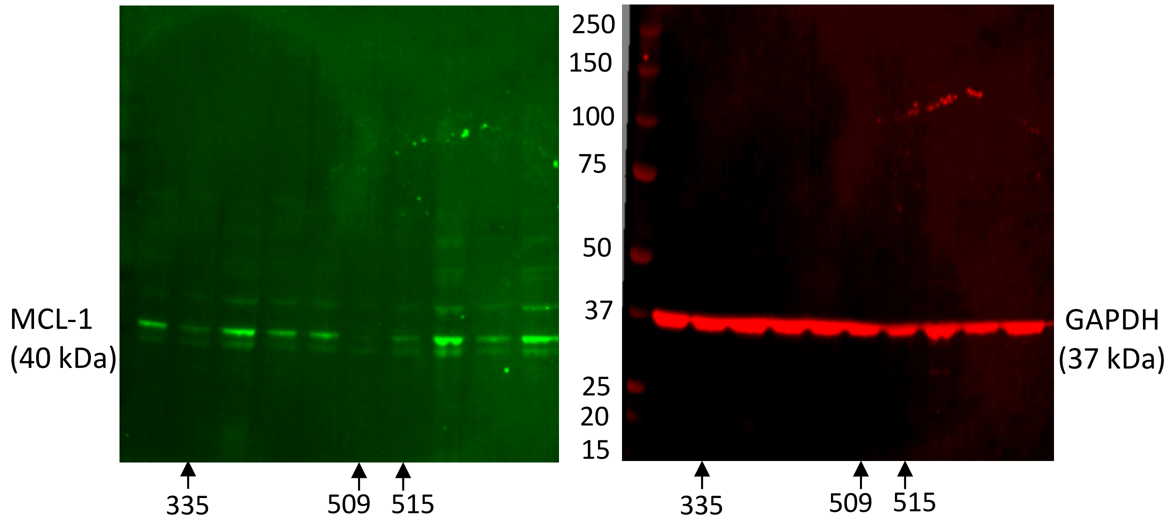

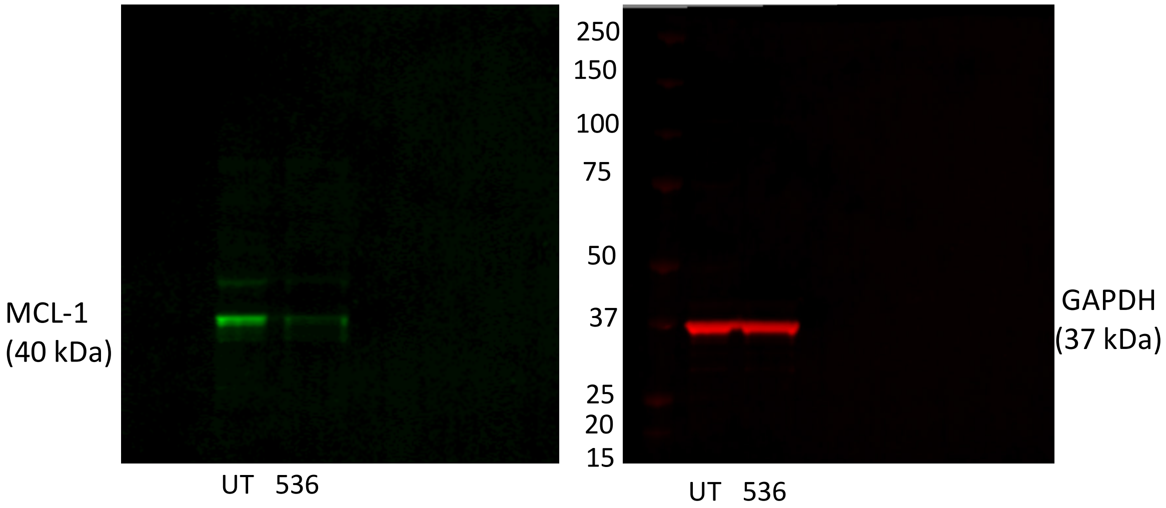

ratio of MCL-1 band to GAPDH band in the same well (raw fluorescence data) before normalization to untreated control.

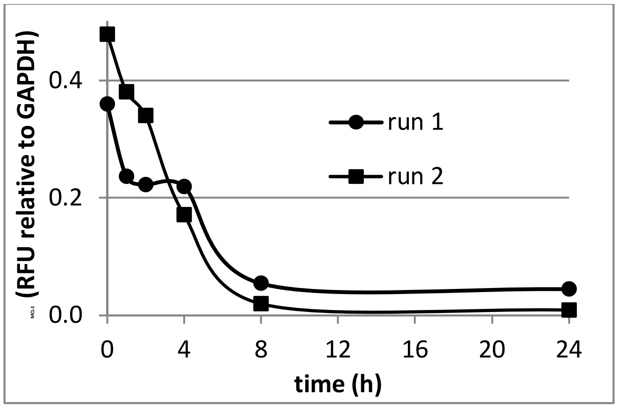

EFFECT OF ROCAGLATES ON cJUN PHOSPHORYLATION (main Fig 6A)

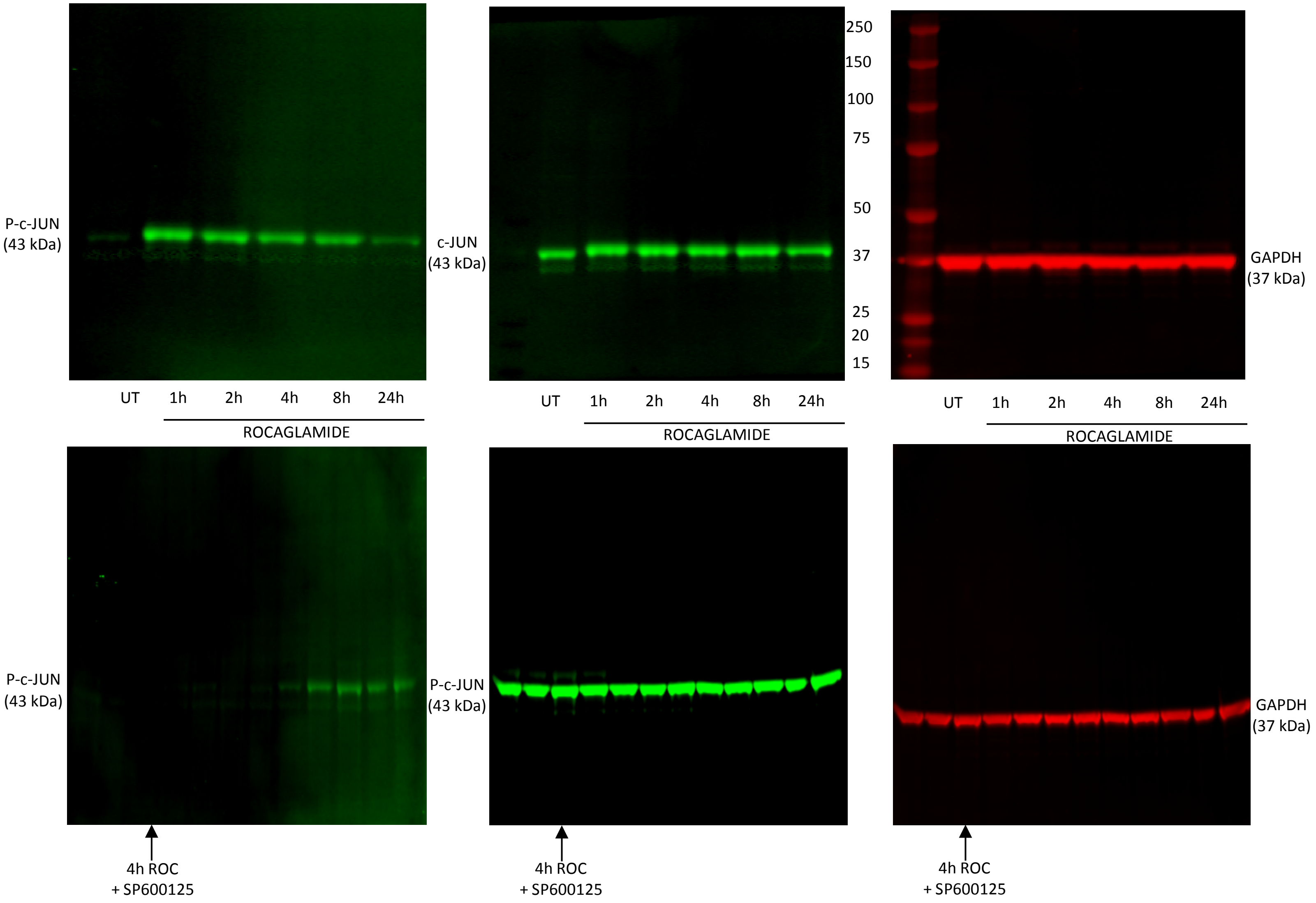

EFFECT OF ROCAGLATES ON cJUN PHOSPHORYLATION (main Fig 6A)

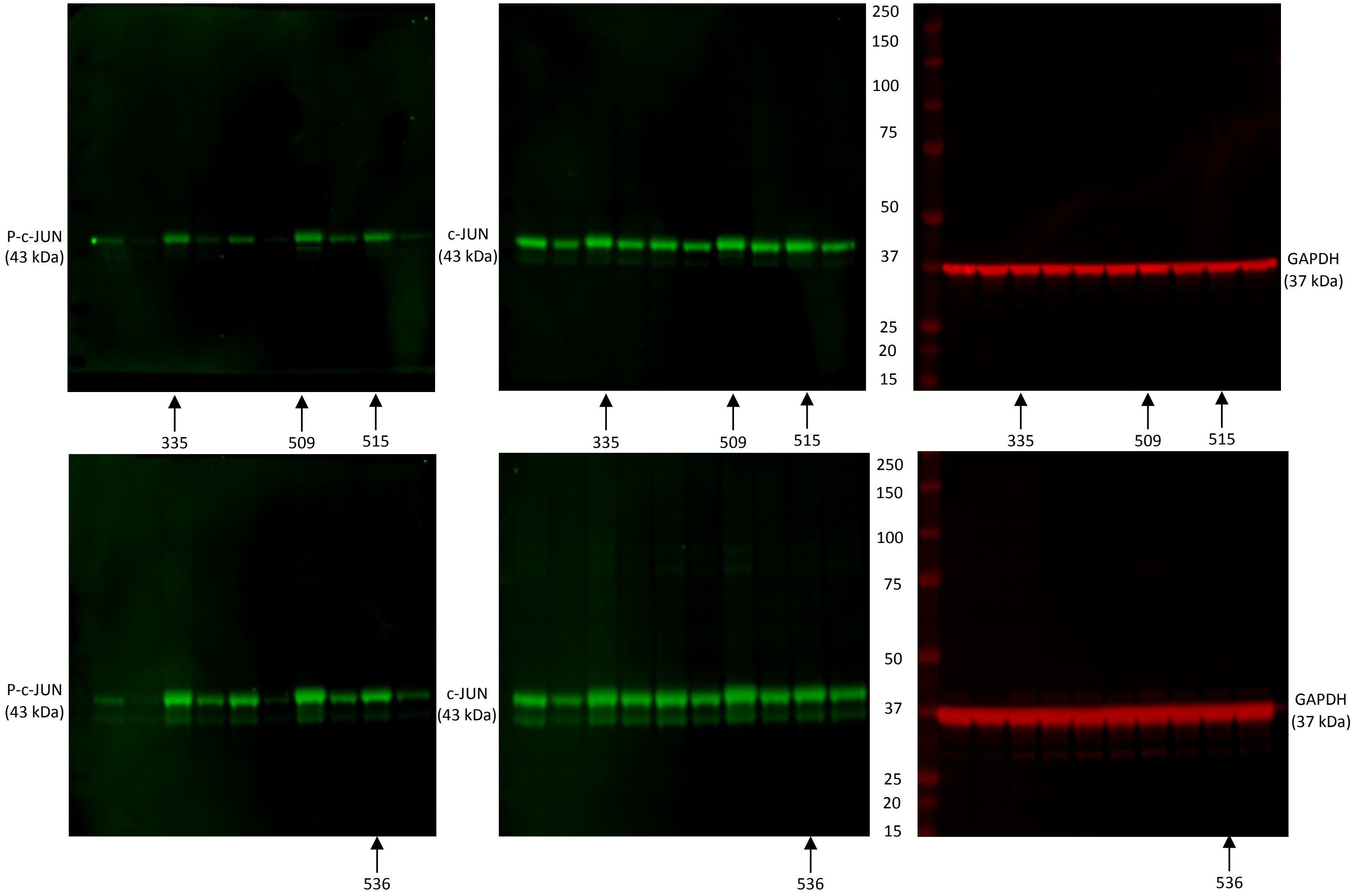

EFFECT OF ROCAGLATES ON ERK PHOSPHORYLATION (main Fig 6C)
(stripped, re-probed for GAPDH)

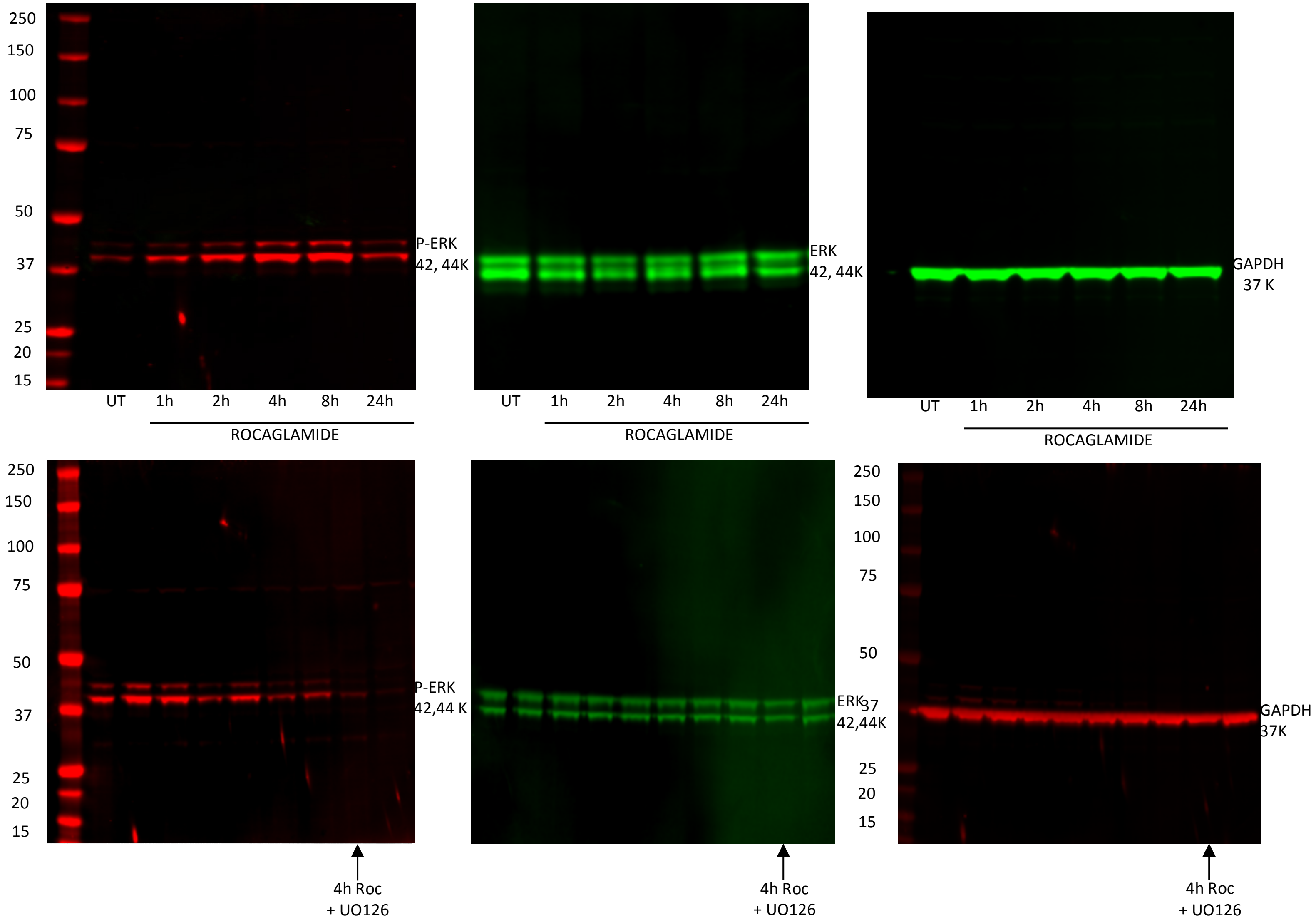

EFFECT OF ROCAGLATES ON ERK PHOSPHORYLATION (main Fig 6C)

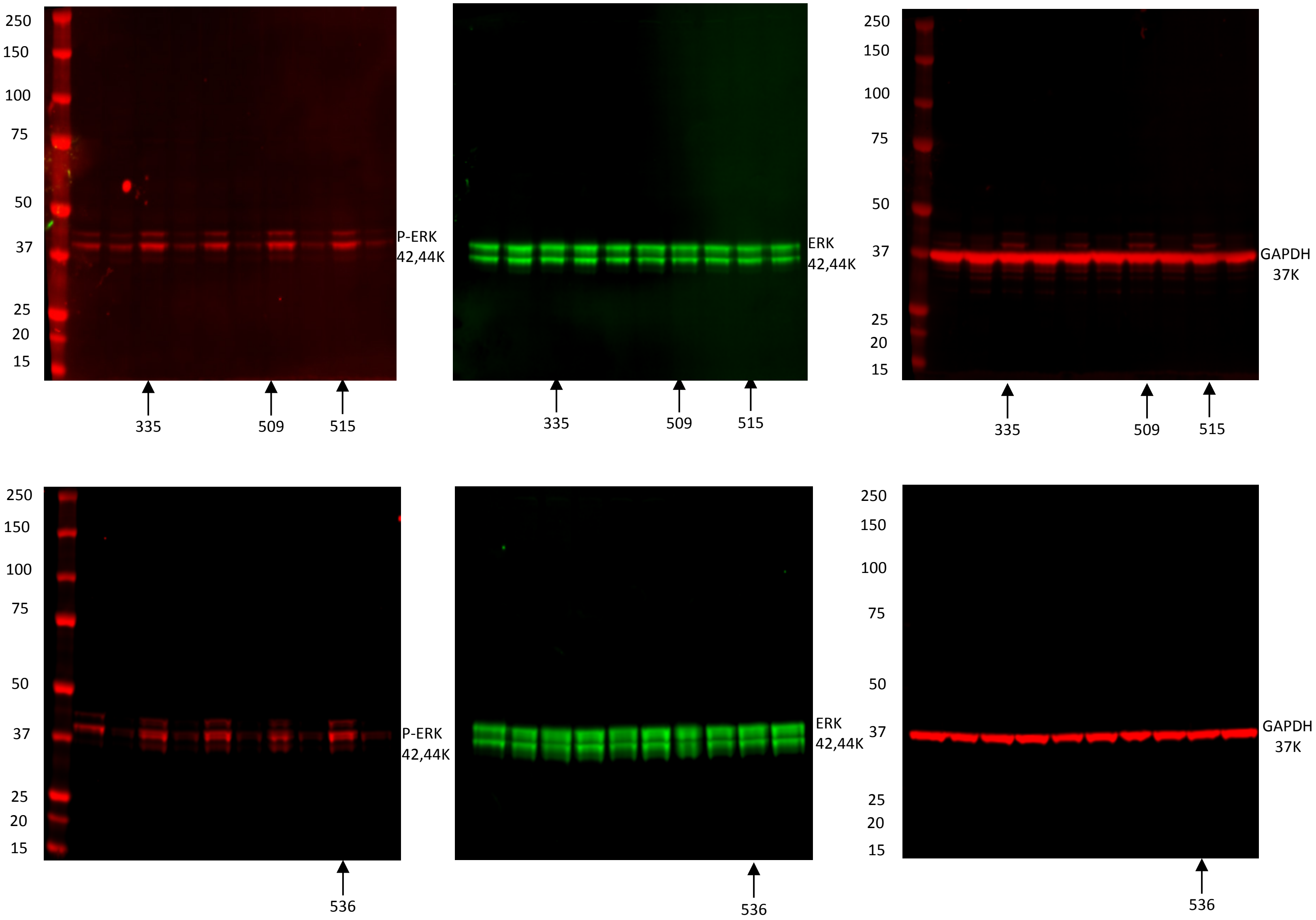

Supplement: Supplementary file 1 — Supplementary Information [file 41598_2018_35908_MOESM1_ESM.pdf]
